# Survey of Physicians’ Perspectives and Knowledge about Diagnostic Tests for Bloodstream Infections

**DOI:** 10.1371/journal.pone.0121493

**Published:** 2015-03-26

**Authors:** Rosemary C. She, Sally Alrabaa, Seung Heon Lee, Meghan Norvell, Andrew Wilson, Cathy A. Petti

**Affiliations:** 1 Department of Pathology, Keck School of Medicine of the University of Southern California, Los Angeles, California, United States of America; 2 Department of Medicine, Morsani School of Medicine, University of South Florida, Tampa, Florida, United States of America; 3 nanoMR Inc., Albuquerque, New Mexico, United States of America; 4 Department of Family and Preventive Medicine, University of Utah, Salt Lake City, Utah, United States of America; 5 HealthSpring Global Inc., Bradenton, Florida, United States of America; Royal Tropical Institute, NETHERLANDS

## Abstract

**Background:**

Physicians rely on blood culture to diagnose bloodstream infections (BSI) despite its limitations. As new technologies emerge for rapid BSI diagnosis, optimization of their application to patient care requires an understanding of clinicians’ perspectives on BSI diagnosis and how a rapid test would influence medical decisions.

**Methods:**

We administered a 26-question survey to practitioners in infectious diseases/microbiology, critical care, internal medicine, and hematology/oncology services in USA and Germany about current standards in diagnosing and treating BSI and a hypothetical rapid BSI test.

**Results:**

Responses from 242 providers had roughly equal representation across specialties. For suspected BSI patients, 78% of practitioners would administer empiric broad spectrum antibiotics although they estimated, on average, that 31% of patients received incorrect antibiotics while awaiting blood culture results. The ability of blood culture to rule in or rule out infection was very/extremely acceptable in 67% and 36%, respectively. Given rapid test results, 60–87% of practitioners would narrow the spectrum of antimicrobial therapy depending on the microorganism detected, with significantly higher percentages when resistance determinants were also tested. Over half of respondents felt a rapid test would be very/extremely influential on clinical practice.

**Conclusions:**

Limitations of blood culture were perceived as a barrier to patient care. A rapid test to diagnose BSI would impact clinical practice, but the extent of impact may be limited by prevailing attitudes and practices. Opportunities exist for interventions to influence practitioners’ behaviors in BSI management particularly with emergence of newer diagnostic tests.

## Introduction

Bloodstream infections remain one of the most common conditions of patients in US hospitals, and rank in the top four most costly conditions [1]. Clinical improvement initiatives on the management of patients with bloodstream infections (BSIs) are under increased scrutiny because BSIs are associated with high rates of morbidity and mortality with roughly half of deaths in U.S. hospitals occurring in patients with sepsis [2]. Advocacy for and adoption of better diagnostic tests to improve patient outcomes are major components of these initiatives, and large international organizations such as the Infectious Diseases Society of America have prioritized this need [3]. Diagnosis of BSI presently relies on blood culture—a method with a relatively long time to result when compared with molecular tests that can provide results for *Clostridium difficile* or respiratory viral infections within 90 minutes. Intuitively, improved, faster diagnostics for BSI should promote better clinical decisions, improve outcomes, and lower costs. In practice, we do not have a clear understanding of the process by which test results influence clinical decisions, and we have little data to support the assertion that rapid tests for BSI alone change physicians’ cognitive and behavioral patterns, leading to improved patient outcomes. On the contrary, clinical practices do not drastically change with mere implementation of new technology for blood pathogen identification, but require additional interventions such as antibiotic stewardship review, or use of electronic “bundles,” without which little effect is seen on patient care [4–7]. In further support of this rapid diagnostic test paradox, a Cochrane review of rapid malaria tests for patients with fever found rapid testing did not improve patient outcomes, had little influence on antibiotic prescriptions, and only reduced antimalarial prescribing patterns when providers believed the test results [8]. We submit that decision analyses of physician practice patterns and attitudes towards BSI diagnostic testing can be instructive and potentially identify factors that undermine or support the introduction of innovative technologies. Knowledge about physicians’ attitudes and behaviors is critical and may provide opportunities to develop targeted policies and build supportive infrastructure for optimal implementation of rapid BSI diagnostics. Through a survey design, we sought to elucidate clinicians’ perspectives on the diagnosis and management of patients with bloodstream infections and ascertain how new diagnostic tests for BSI would influence medical decisions and potentially improve patient care.

## Methods

Clinicians with specialties in infectious diseases/microbiology, critical care, hematology/oncology and general medicine/hospitalist services in the United States and Germany were invited to participate in the survey. The survey was conducted from March 2013 to January 2014. The 26 question survey instrument was developed by a team of infectious diseases specialists, microbiologists, pathologists, and biostatisticians, and pilot tested by infectious diseases specialists from several countries (S1 Questionnaire). The questionnaire collected information on basic demographic data (specialty, years in practice, country, patient volumes), respondents’ knowledge of performance metrics for culture-based tests, current practices of care in diagnosing and treating BSI, perceptions about barriers to effective care, and hypothetical use of a rapid BSI test. The survey was administered in academic settings with IRB exemption (University of Southern California) or approval (University of South Florida). Practitioners were recruited randomly at departmental meetings and by email solicitation to complete a written survey. Responses were anonymous. The survey was also administered in non-academic settings via market research tools in collaboration with nanoMR, Inc. (Albuquerque, NM), a company developing a sample preparation product for rapid detection of infectious diseases. Given the mechanisms of survey distribution, the number of respondents declining to participate could not be ascertained.

To classify physicians with multiple specialties, a hierarchy was created to partition primary specialty categories: (1) if any of their specialties was infectious disease or microbiology, they were classified as “infectious diseases/microbiology,” (2) else if they had critical care as a specialty they were classified as “critical care,” else if they were internal medicine specialists or hospitalists they were classified as “internal medicine/hospitalist,” else, finally, they were classified as “other.” For example, someone with all specialties would be classified as “infectious disease/microbiology.” For statistical analysis, we used general linear models to investigate relationships between continuous variables and Chi-square or Fisher’s exact tests to examine relationships between categorical variables. Cronbach’s alpha was used to assess internal consistency of clusters or theoretically related test questions. All calculations were performed using SAS software, Version 9.3 of the SAS System Copyright © 2011 SAS Institute Inc., Cary, NC, USA. Results were considered statistically significant if p<0.05.

## Results

### Participant demographics

Responses were obtained from 242 participants (ages 26–71, mean 40.9, median 40.0 years) consisting of 228 M.D. physicians, 8 D.O. physicians, one physician assistant, three nurse practitioners, and two infection prevention practitioners. Specialties represented included 78 (32.2%) internal medicine/hospitalist, 90 (37.2%) infectious diseases/microbiology, 60 (24.8%) critical care, 4 (1.7%) hematology/oncology, and 10 (4.1%) other specialties. Years in medical practice ranged from less than one to 43 years (mean 12.6, median 10 years). Seventy-six practiced in Germany and 166 practiced in the U.S.A.

### Perceptions, practices and knowledge of present standard of care

**Fig 1:**
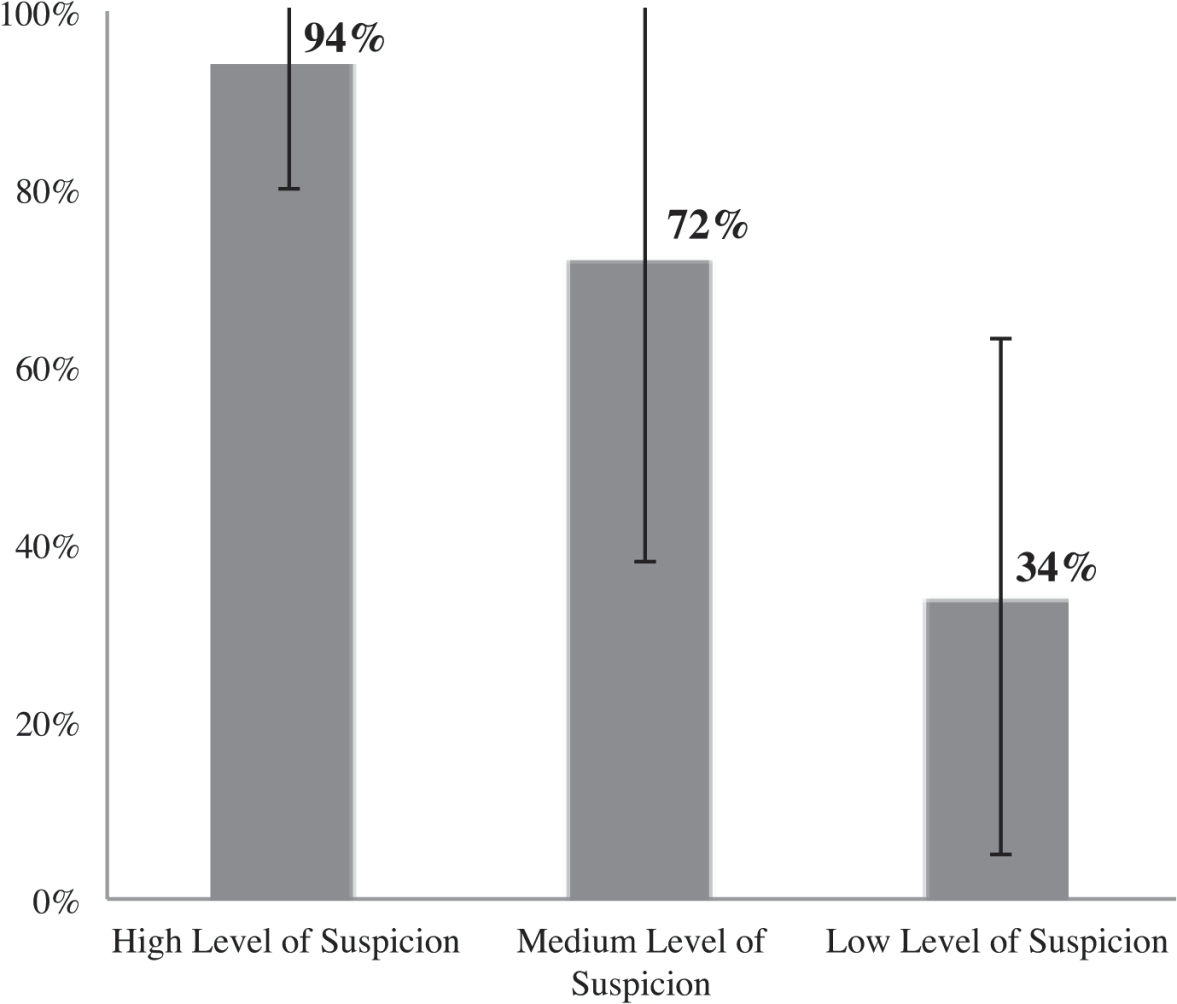
Percentage of practitioners that would administer empirical therapy stratified by patient risk for BSI (Q10). Mean values are shown with one standard deviation error bars.

The participants consulted on an average of 186 hospital inpatients per month (range, 10–800). Respondents perceived febrile neutropenic patients as the likeliest to have BSI, followed by patients in critical/intensive care and those with indwelling devices. When evaluating patients with suspected BSI, practitioners responded that they would administer empirical broad or narrow spectrum antibiotics to 78% and 13% of patients on average (Q11), respectively, and in such cases 97% would “always” or “almost always” order a blood culture test. Practitioners in the United States reported ordering blood cultures for patients with suspected bloodstream infections more frequently than compared to practitioners in Germany (p < 0.0027). Fig. 1 demonstrates reported behaviors for use of empirical therapy for patients with high, medium and low suspicion for BSI. Perceptions about the performance characteristics for blood cultures were investigated on a 5 point Likert scale (Table 1). Sixty-seven percent of respondents rated the ability of blood cultures to rule in an infection as very/extremely acceptable, and only 36% rated its ability to exclude infection as very/extremely acceptable. Notably, turn-around-time of blood culture was rated very/extremely acceptable by only 22% of practitioners. We assessed practitioners’ knowledge of average time to result for positive blood cultures and wide ranges were reported (Table 2). No statistical differences were observed among responses based on specialty. Overall, practitioners responded that from the time of blood collection, Gram stains were reported from instrument signaled blood culture bottles within 12.4 hours (SD ±9.6), and average time to preliminary identification of the pathogen was estimated to be 26.5 hours (±14.1). When responses were compared based on country of origin, German practitioners were more likely to report shorter turn-around-times for laboratory processes as compared with those responses of USA practitioners (Table 2).

**Table 1:** Practitioners’ Perceptions of Blood Culture Performance—Number and Percentage Finding Metric Extremely or Very Acceptable on 5-point Likert Scale.

	**Specialty**					
	**All**	**ID/Microbiology**	**Gen MedHospitalist**	**Critical Care**	**Other**	
**Perception of Blood Culture Performance**	**N (%)**	**n (%)**	**n (%)**	**n (%)**	**n (%)**	***P* Value**
**Ability to rule inan infection**	163 (67)	59 (66)	55 (71)	39 (65)	10 (71)	0.888
**Ability to rule outan infection**	87 (36)	37 (41)	30 (39)	15 (25)	5 (36)	0.212
**Time to result**	53 (22)	27 (30)	15 (19)	10 (17)	1 (7)	0.099
**Cost**	85 (37)	33 (41)	24 (31)	23 (40)	5 (42)	0.578

**Table 2:** Practitioner Knowledge of Turn-Around-Time for Positive Blood Cultures and Attitude Toward Turn-around-Time Requirement for Hypothetical Rapid Test.

		**Country of Origin**			
		**All**	**Germany**	**USA**	
	**Time to Result by Laboratory Process**	**Mean (SD) Hours**	**Mean (SD) Hours**	**Mean (SD) Hours**	***P* Value**
**Knowledge**	**Time to Gram stain result from instrument signaled bottle**	12.4 (9.6)	11.0 (9.5)	13.1 (9.6)	0.116
	**Time to preliminary microorganism identification**	26.5 (14.1)	22.3 (15.0)	28.5 (13.3)	0.002
	**Time to final identification and antimicrobial susceptibility testing**	48.3 (21.6)	39.0 (22.9)	52.8 (19.4)	<0.0001
**Attitude**	**Maximum turn-around-time rapid test provides no additional value**	4.7 (2.2)	4.4 (2.0)	4.9 (2.2)	0.071

**Fig 2:**
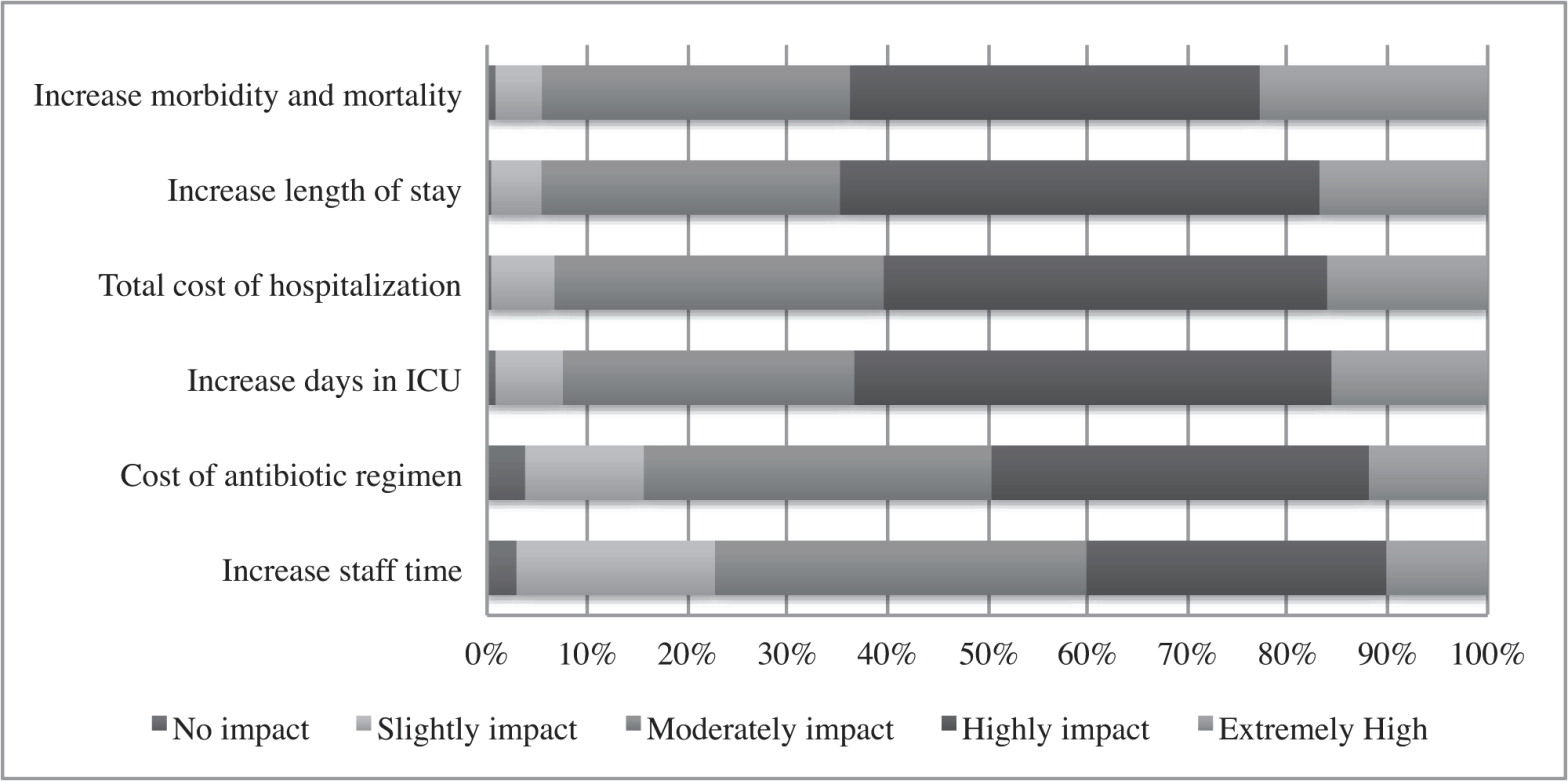
Perceived effect of suboptimal antimicrobial therapy. Practitioners were asked for their perceptions about how incorrect or suboptimal antimicrobial therapy influenced clinical and financial factors in patient care.

A mean of 31% of practitioners believed patients are treated with incorrect antibiotics while awaiting blood culture results, and 74% reported a financial cost from relying on blood culture results that can potentially lead to a delay in clinical decisions. Fig. 2 demonstrates practitioners’ perspectives on how incorrect or suboptimal antimicrobial regimens may affect the course of a patient’s hospitalization in terms of outcomes and cost.

### Perceptions of hypothetical rapid test’s influence on prescribing practices

**Fig 3:**
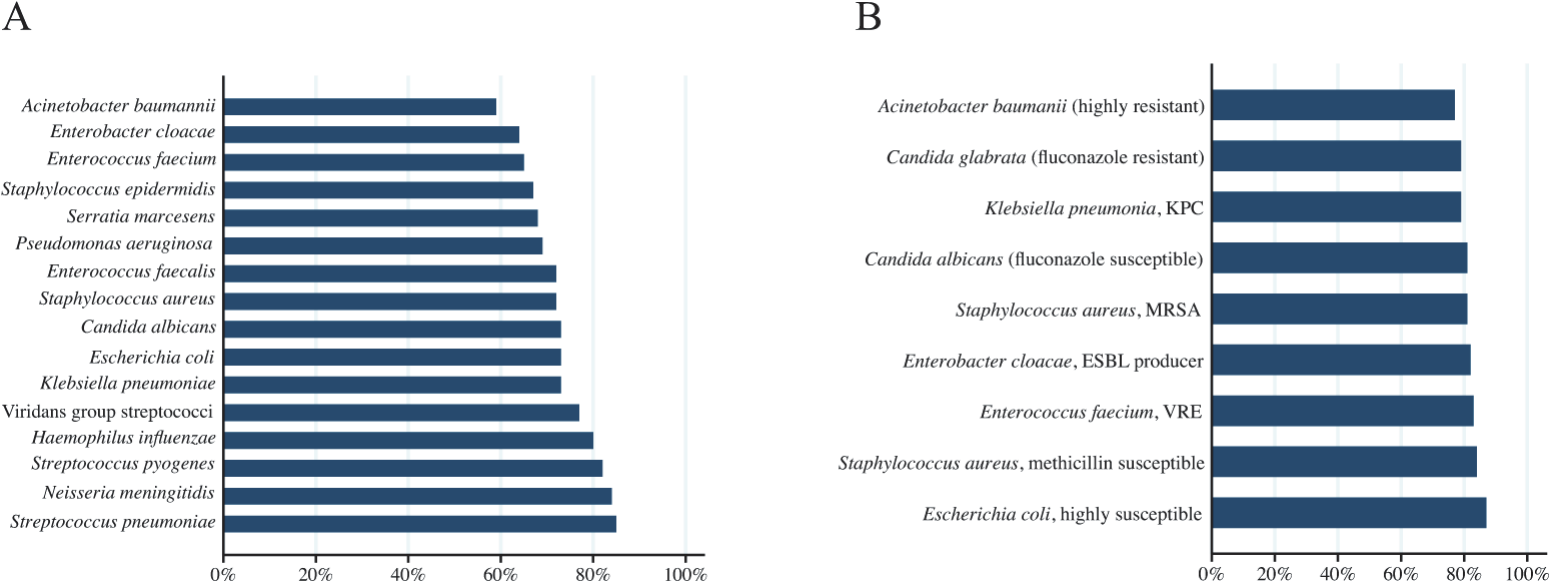
Influence of test results on antimicrobial therapy. Shown in Panel A are the percentages of respondents that would narrow empirical antimicrobial therapy based on rapid test identification result is shown. Panel B displays the percentage of respondents that would change empirical antimicrobial therapy based on rapid test identification result if preliminary susceptibility results were included.

German and USA practitioners’ responded that results from a hypothetical rapid test would provide additional value to clinical care when reported within 5 hours from blood collection (Table 2). Overall, more than 50% of practitioners rated that a rapid test would be very/extremely influential on clinical practice indicators such as antibiotic consumption, antimicrobial resistance, and patient morbidity/mortality with no significant differences among specialties (Table 3). Fig. 3A and 3B graphically display practitioners reported behaviors when given the pathogen identification from a hypothetical rapid test with or without susceptibility results. Between 60 and 85% of participants would narrow the spectrum of antimicrobial therapy based on pathogen identification alone, and 77–87% would narrow/change antimicrobials when susceptibility results also were provided. The type of organism, i.e. viridans group streptococcus, *Streptococcus pneumoniae*, *Neisseria meningitidis*, *Streptococcus pyogenes*, and *Haemophilus influenzae*, had a large effect on practitioners’ likelihood of narrowing antibacterial therapy as compared to the other group of listed pathogens (p < 0.001). No statistical differences in reported behavior were identified by practitioner specialty or country of origin. The only statistically significant correlation between respondents administering broad or narrow spectrum therapy (Q11) and their intent to narrow therapy based on a hypothetical rapid test result (Q15) was a negative correlation between use of broad spectrum antibiotics and narrowing therapy based on a result of *S*. *aureus* (Spearman Rho = -0.171; p = 0.0282). Reported behaviors changed significantly when the hypothetical test was reported with and without susceptibility results (Table 4).

**Table 3:** Practitioner Perceptions of Hypothetical Rapid Test on Clinical Practice—Number and Percentage Finding Test Very/Extremely Influential on 5-point Likert Scale.

	**Specialty**					
	**All**	**ID/ Microbiology**	**Gen Med Hospitalist**	**Critical Care**	**Other**	
**Perceptions**	**N (%)**	**n (%)**	**n (%)**	**n (%)**	**n (%)**	***P* Value**
**Reduce overall antibiotic consumption**	141 (60)	54 (62)	46 (61)	35 (58)	6 (46)	0.729
**Decrease emergence of antimicrobial resistance**	138 (60)	50 (58)	39 (52)	41 (68)	8 (62)	0.289
**Reduce intensity of health-care utilization (i.e. imaging studies, other blood tests, specialty consults, etc.)**	109 (46)	36 (41)	38 (51)	32 (53)	3 (23)	0.149
**Decrease patient morbidity and mortality**	125 (53)	43 (49)	43 (58)	33 (56)	6 (46)	0.653
**Reduce infection transmission to other susceptible patients**	96 (41)	37 (43)	32 (44)	23 (38)	4 (29)	0.727
**Reduce overall costs of hospitalization**	103 (44)	35 (40)	32 (43)	29 (48)	7 (58)	0.572
**Payer satisfaction**	80 (34)	26 (30)	28 (37)	21 (35)	5 (42)	0.702
**Patient satisfaction**	100 (43%)	37 (43%)	33 (44%)	26 (43%)	4 (31%)	0.883

**Table 4:** Number and Percentage of Practitioners’ Responding that They Would Change Empiric Antimicrobial Therapy Based on Hypothetical Rapid Test Result.

**Result Reported:**	**Number (%) Responding “Yes” to Change Therapy**	**Result Reported:**	**Number (%) Responding “Yes” to Change Therapy**	***P* Value for proportion difference** ^a^
**Microorganism Only**		**Microorganism with Susceptibility**		
*Staphylococcus aureus*	162/224 (72)	*Staphylococcus aureus*, methicillin susceptible	189/225 (84)	p = 0.0027
*Staphylococcus aureus*	162/224 (72)	*Staphylococcus aureus*, methicillin resistant	189/232 (82)	p = 0.0204
*Enterococcus faecium*	136/208 (65)	*Enterococcus faecium*, vancomycin resistant	190/228 (83)	p < 0.0001
*Escherichia coli*	154/212 (73)	*Escherichia coli*, “highly susceptible”	199/229 (87)	p = 0.0002
*Klebsiella pneumoniae*	158/216 (73)	*Klebsiella pneumoniae*,	173/219 (79)	p = 0.1528
*Enterobacter cloacae*	125/195 (64)	*Enterobacter cloacae*, ESBL producer	186/228 (82)	p < 0.0001
*Acinetobacter baumannii*	116/195 (60)	*Acinetobacter baumannii*, “highly resistant”	169/220 (77)	p = 0.0001
*Candida albicans*	156/214 (73)	*Candida albicans*, fluconazole susceptible	175/217 (81)	p = 0.0567

^a^ Two-sample test of proportions.

### Attitudes and behaviors towards adoption of rapid test

Practitioners reported that the most influential decision-makers for test adoption were MD or PhD laboratory medical directors and laboratory administrators. The highest rated obstacle for test adoption for USA practitioners was lack of evidence for clinical utility (Table 5). On a 4 point scale from earliest/risk taker to late adopter/wait until well tested and published, those responding as being the earliest adopters were those with the most years in clinical practice (p<0.0001). The maximum price point (on a scale from $100-$800) at which most practitioners would pay was $100-$200 (84%) for an adjunct rapid test for blood culture, and $100-$200 (72%) for a rapid stand-alone test for BSI.

**Table 5:** Attitudes about Barriers to Adoption for Rapid Test—Number and Percentage Who Agree/Strongly Agree with Each Statement.

	**Country of Origin**			
	**All**	**Germany**	**USA**	
**Attitudes**	***N* (%)**	***n* (%)**	***n* (%)**	***P* Value**
**Physician uncertainty of using new technology in clinical practice**	132 (57)	26 (34)	106 (68)	<0.0001
**Cost of test**	178 (77)	56 (73)	122 (79)	0.4085
**Lack of evidence for clinical utility**	188 (81)	53 (70)	135 (87)	0.0021
**No reimbursement code or payer concerns**	154 (68)	47 (62)	107 (70)	0.2302

## Discussion

We sought to understand physician perceptions and behaviors with current standards of care for BSI and with adoption of a hypothetical rapid BSI test. To our knowledge, this survey is the first to extensively investigate practitioners’ attitudes about routine blood cultures and the comparative value of rapid testing in terms of patient management, hospital quality measures, and financial costs. With the high mortality rates associated with sepsis, it is not surprising that broad spectrum empirical antimicrobial therapy was reported as an extremely common practice (almost 80%) among respondents in the survey. While nearly all respondents would order a blood culture in patients suspected of having BSI, they were generally dissatisfied with the performance of blood culture—an observation reflected in their survey responses about blood culture’s sensitivity, negative predictive value, and time to result. Limitations of blood culture were perceived as a barrier to effective care, and most believed that a rapid test would improve outcome measures.

Interestingly, attitudes about blood culture did not necessarily reflect actual performance measures as demonstrated in peer-reviewed literature. For example, practitioners reported blood culture sensitivity of 67%, but numerous publications demonstrate that blood culture detects >90% of all bloodstream infections and lower sensitivity is often “iatrogenic” [9]. Additionally, in this survey, practitioners’ abilities to estimate mean times to report laboratory results for Gram stain, preliminary identification, and susceptibility testing were variable. Per the literature, instruments signal blood bottles for Gram positive microorganisms within 13–21 hours and for Gram negative microorganisms within 8–18 hours [10–12]. Furthermore, with conventional methods and/or MALDI-TOF, most identification and susceptibility results can be available within 4 to 30 hours after the initial Gram stain result [13–16]. Practitioners’ overestimation of time to result could be explained by health system differences in laboratory resources (e.g., off-site lab, 24 hour lab staffing), gaps in practitioners’ knowledge, lack of optimization of turn-around-times with current laboratory budgets, or delayed communication of results. Specifically, the significant differences in times to preliminary and final results for USA and Germany most likely can be explained by Germany’s widespread adoption of MALDI-TOF as an identification method from instrument signaled blood culture bottles. In fact, a 2013 European survey about blood culture testing identified inadequate training of personnel, off-site location of laboratories, provider time constraints, and cost as barriers to ordering blood cultures [17]. Factors independent of analytical performance of blood culture may contribute to negative perceptions and behaviors, and an opportunity exists to develop interventions and a more supportive infrastructure to optimize the value of any BSI test regardless of its speed and accuracy. Practitioners’ attitudes about blood culture testing may serve as a barrier to its effective use, but the responses suggest that changes, other than adoption of a rapid test, may be necessary to demonstrate comparative effectiveness.

Despite the lack of consistency of responses for conventional tests, practitioners from any specialty or country felt that a rapid BSI test would need to produce results within approximately 5 hours to provide additional value to conventional tests. This perception may be influenced by many factors including typical dosing intervals of antibacterial agents, average time to Gram stain results, and by a commonly accepted practice that patients treated with ineffective antimicrobial therapy within the first 6 hours of sepsis have a 7.6% reduction in survival [18]. Practitioners also felt that a rapid test would have a positive effect on clinical practice measures. Yet, when a result from a hypothetical test to detect pathogens directly from peripheral blood was reported within hours, it was not abundantly clear from this survey that early pathogen detection would necessarily lead to widespread changes in clinical practice. Most practitioners reported that they would change empirical antimicrobial therapy based on results of a rapid test, but up to 40% of practitioners responded that they were uncertain or would not change behavior. Interestingly, compared to all other pathogens, practitioners were more likely to report that they would alter empirical therapy for viridans group streptococci, *H*. *influenzae*, *S*. *pyogenes*, *N*. *meningitidis*, and *S*. *pneumoniae*—a group of pathogens generally well-recognized to be susceptible to most antibacterial drugs. For rapid test results with staphylococci, enterococci, and gram-negative bacilli (the most common pathogens causing BSI), a significantly lower percentage of respondents reported that they would change empirical regimens. When susceptibility results were reported in descriptive terms, differences in behavior towards changing antibacterial therapy were observed. This finding may reflect a limitation in our study design, potentially providing an “ideal” result implying all possible and known resistance markers were tested. On the other hand, practitioners’ knowledge of resistance mechanisms may be inadequate, and descriptions may help prompt changes in prescribing behavior. In fact, several studies have demonstrated that interventions such as persuasive feedback, antimicrobial stewardship, restriction of antibiotic use and pharmacy led initiatives influence physician antibiotic prescribing practices [19]. Differences in responses for pathogens with extended-spectrum beta-lactamase (ESBL) versus *Klebsiella pneumoniae* carbapenemase (KPC) mechanisms of resistance could reflect greater familiarity with treatment options for ESBL producers or reflect practitioners’ attitudes about the greater complexity in managing patients with carbapenemase-producing organisms. A hypothetical rapid test that reports a pathogen with or without a few resistance markers does not capture the highly nuanced field of resistance mechanisms, and the results may not produce the desired effect in changing prescribing behaviors. In summary, these responses suggest that further study is necessary to more fully elucidate cognitive and behavioral patterns among practitioners following receipt of pathogen specific and susceptibility test results. Also, prospective studies are necessary to explore the value of more interpretative, descriptive reporting of test results from either conventional or nucleic acid based tests that may assist the practitioner with antimicrobial selection.

Approximately 75% of respondents perceived that delayed diagnosis inherent to the process of blood culturing was associated with a financial cost. Also, respondents estimated that nearly one-third of patients were treated with incorrect antibiotics while awaiting blood culture results, and the delay in initiating effective antimicrobial therapy was perceived to adversely influence patient outcome measures. In this context, it is not surprising that practitioners reported widespread use of empirical antimicrobial therapy including for those patients with low likelihood of BSI. Indeed, studies have shown poor outcomes, particularly in the intensive care setting, from inadequate antimicrobial therapy and multidrug-resistant organisms causing BSI [20, 21]. With blood culture still the mainstay of BSI diagnosis, it is well-accepted that identification of the pathogen is delayed and clinical decisions must be made before results are available, precipitating the widespread practice of antimicrobial empiricism. These perceptions and behaviors of empiricism, however, may undermine the value of a rapid diagnostic test in influencing changes in prescribing patterns. Herein lies an opportunity to better understand the conditions that perpetuate empirical use of antimicrobials, identify cognitive triggers among practitioners that promote this practice even in the face of a rapid, accurate test result, and eventually develop interventions to modify behavior.

Several limitations existed in this study. We do not know the number or type of respondents unwilling to participate in the survey that could have caused sample bias. For the hypothetical rapid test scenarios, we specifically excluded patient clinical information to reduce time to complete the survey. The lack of ancillary data may have contributed to uncertainty of some responses. For example, regardless of pathogen identified, practitioners may be less likely to change antibacterial therapy for patients with concurrent infections such as wound infections or osteomyelitis that require continued broad spectrum antimicrobial therapy or for patients already improving on current antibacterial regimens. Finally, we did not design follow-up questions to query practitioners about how they would alter therapy following the rapid test result that could have elucidated their predilections in prescribing behaviors and better understand antimicrobial utilization.

In recent years, new technologies have been introduced into laboratories, and their evaluations have primarily focused on technical performance (speed and accuracy), clinical utility, and financial drivers (laboratory, hospital and payer). The ability of a test to influence clinical decisions and change practice also depends on practitioners’ perceptions and behaviors in ordering tests and acting on test results. As we have shown in this survey study, practitioners’ perceive that diagnosis of bloodstream infection remains suboptimal and better test modalities can help minimize adverse outcomes and decrease hospital costs. But practitioners’ responses revealed a gap in our understanding of the value drivers for changing practice patterns for patients with BSI, and this knowledge gap could undermine the comparative effectiveness of adoption of new tests. Assessment of physician perceptions and behaviors related to diagnostic tests for BSI management are vital to creating the best implementation strategies for BSI testing and optimizing their value for effective care. Development of faster and better BSI tests is important, but as this survey demonstrated, the introduction of superior tests may need to be coupled with systematic efforts to influence clinical decisions and outcomes.

## Supporting Information

S1 Datamap Key to survey response tabulation. This datamap demonstrates the values assigned to survey responses as recorded in the dataset.
(XLSX)

S1 Dataset Dataset of questionnaire responses. The tabulation of responses to each survey question is shown. Values for responses were assigned as outlined in the datamap.
(PDF)

S1 Questionnaire Survey questionnaire. This is the 26 question questionnaire administered in this study.
(DOCX)
