# Supplementary material for: Survey of Physicians’ Perspectives and Knowledge about Diagnostic Tests for Bloodstream Infections
Source: PLoS One. 2015 Mar 26;10(3):e0121493. doi: 10.1371/journal.pone.0121493 (PMC4374856; doi:10.1371/journal.pone.0121493)
Supplement: S1 Dataset — The tabulation of responses to each survey question is shown. Values for responses were assigned as outlined in the datamap. (PDF) [file pone.0121493.s002.pdf]

ID	Q1	Q2_1	Q3_1	Q4	Q4_5_othe	Q5_1	Q5_2	Q5_3	Q5_4	Q5_5	Q5_6	Q5_6_other	Q5Quota_	Q5Quota_	Q5Quota_3	Q6_1	Q7_1	Q8_1	Q8_2
1	USA	31	8	1		0	1	0	0	0	0			1		30	10	3	5
2	USA	51	20	1		0	0	0	1	0	0				1	300	15	2	4
3	USA	36	7	1		0	1	0	0	0	0			1		700	25	2	5
4	USA	32	6	1		1	0	0	0	0	0		1			70	10	2	3
5	USA	58	26	1		0	0	0	1	0	0				1	560	10	3	4
6	USA	46	17	1		0	0	0	1	0	0				1	470	35	3	4
7	USA	56	22	1		0	0	0	1	0	0				1	400	15	3	3
8	USA	41	17	1		0	1	0	0	0	1	Geriatrics and nephrology		1		150	65	5	3
9	USA	53	24	1		0	1	0	0	0	0			1		251	35	3	2
10	USA	32	7	1		0	1	0	0	0	0			1		70	10	2	4
11	USA	42	13	1		1	0	0	0	0	0		1			50	30	2	3
12	USA	56	17	1		0	0	0	1	0	0				1	275	10	5	4
13	USA	29	3	1		0	1	0	0	0	0			1		200	25	2	2
14	USA	33	7	1		0	1	0	1	0	1	Pediatrics		1	1	340	10	3	3
15	USA	41	11	1		0	0	0	1	0	0				1	300	65	4	5
16	USA	44	17	1		1	0	0	0	0	0		1			60	20	3	5
17	USA	52	26	1		0	0	0	1	0	0				1	120	10	4	3
18	USA	49	23	1		0	0	0	1	0	0				1	100	40	2	3
19	USA	33	7	1		0	1	0	0	0	0			1		80	10	5	4
20	USA	41	11	1		0	0	0	1	0	0				1	100	70	5	4
21	USA	50	21	1		1	0	0	0	0	0		1			150	15	5	4
22	USA	48	14	1		0	1	0	0	0	0			1		300	10	4	5
23	USA	28	4	1		0	1	0	0	0	0			1		140	10	3	4
24	USA	30	7	1		0	1	0	0	0	0			1		400	30	4	5

ID	Q1	Q2_1	Q3_1	Q4	Q4_5_othe	Q5_1	Q5_2	Q5_3	Q5_4	Q5_5	Q5_6	Q5_6_other	Q5Quota_	Q5Quota_	Q5Quota_	Q6_1	Q7_1	Q8_1	Q8_2
25	USA	45	20	1		0	0	0	1	0	0				1	200	50	3	4
26	USA	33	5	1		0	1	0	0	0	0			1		300	25	4	4
27	USA	36	7	1		0	0	0	1	0	0				1	50	70	4	3
28	USA	27	2	1		0	0	1	0	0	0		1			80	15	4	4
29	USA	31	7	1		0	1	0	0	0	0			1		400	40	5	5
30	USA	37	6	1		0	0	0	1	0	0				1	350	20	5	5
31	USA	40	7	1		1	0	0	0	0	0		1			300	20	3	3
32	USA	35	8	1		0	1	0	0	0	0			1		150	20	2	5
33	USA	33	7	1		0	1	0	0	0	0			1		300	80	5	3
34	USA	31	7	1		0	1	0	1	0	0			1	1	75	40	3	4
35	USA	26	1	1		0	1	0	0	0	0			1		100	25	4	5
36	USA	61	28	1		1	0	0	0	0	1	critical care/cardiology	1			50	10	5	4
37	USA	38	10	1		1	0	0	0	0	0		1			150	10	4	4
38	USA	52	19	1		0	0	0	1	0	0				1	30	15	3	3
39	USA	52	20	1		0	1	0	0	0	0			1		200	15	4	4
40	USA	32	5	1		0	0	0	1	0	0				1	100	50	3	4
41	USA	41	13	1		0	0	0	1	0	0				1	80	20	4	4
42	USA	53	30	1		1	0	0	0	0	0		1			250	15	5	4
43	USA	50	24	1		0	0	0	1	0	0				1	150	20	3	3
44	USA	53	22	1		0	0	0	1	0	0				1	250	50	4	4
45	USA	34	10	1		1	0	0	0	0	0		1			100	20	5	3
46	USA	42	12	1		0	1	0	1	0	0			1	1	600	20	4	3
47	USA	34	9	1		0	1	0	0	0	0			1		100	10	4	3
48	USA	30	8	1		0	1	0	0	0	0			1		60	15	2	4
49	USA	56	30	1		0	0	0	1	0	0				1	25	12	4	4
50	USA	31	5	1		0	1	0	0	0	0			1		400	10	3	4

ID	Q1	Q2_1	Q3_1	Q4	Q4_5_othe	Q5_1	Q5_2	Q5_3	Q5_4	Q5_5	Q5_6	Q5_6_other	Q5Quota_1	Q5Quota_2	Q5Quota_3	Q6_1	Q7_1	Q8_1	Q8_2
51	USA	38	6	1		0	0	0	1	0	0				1	100	50	3	4
52	USA	38	8	1		0	0	0	1	0	0				1	60	20	3	3
53	USA	36	10	1		0	0	0	1	0	0				1	100	50	3	3
54	USA	54	21	1		0	0	0	1	0	0				1	350	55	5	5
55	USA	29	5	1		0	1	0	0	0	0			1		100	20	4	3
56	USA	40	12	1		0	1	0	0	0	0			1		200	40	5	3
57	USA	34	8	1		1	0	1	0	0	0		1			300	10	5	4
58	USA	33	8	1		1	0	0	0	0	0		1			60	15	4	3
59	USA	53	21	1		0	0	0	1	0	0				1	800	60	4	4
60	USA	50	16	1		1	0	0	0	0	0		1			450	12	4	3
61	USA	54	21	1		0	0	0	1	0	0				1	220	40	4	4
62	USA	52	35	1		0	0	0	1	0	0				1	125	15	4	5
63	USA	35	9	1		1	0	0	0	0	0		1			250	20	5	4
64	USA	54	21	1		0	0	1	0	0	0		1			160	20	3	3
65	USA	52	22	1		1	0	0	0	0	0		1			200	10	3	2
66	USA	44	15	1		0	0	1	0	0	0		1			35	12	4	3
67	USA	58	28	1		1	1	0	0	0	1	Emergency Medicine	1	1		60	15	4	3
68	USA	54	29	1		1	0	0	0	0	0		1			100	20	3	4
69	USA	42	12	1		1	0	0	0	0	0		1			130	10	5	3
70	USA	44	15	1		1	0	0	0	0	0		1			100	25	2	4
71	USA	64	30	1		1	0	0	0	0	0		1			350	20	3	3
72	USA	55	26	1		0	1	0	0	0	0			1		50	25	3	3

ID	Q1	Q2_1	Q3_1	Q4	Q4_5_othe	Q5_1	Q5_2	Q5_3	Q5_4	Q5_5	Q5_6	Q5_6_other	Q5Quota_	Q5Quota_	Q5Quota_	Q6_1	Q7_1	Q8_1	Q8_2
73	USA	42	12	1		1	0	0	0	0	0		1			400	25	4	4
74	USA	45	11	1		1	0	0	1	0	0		1		1	250	20	5	5
75	USA	54	28	1		0	0	0	1	0	0		0	0	1	45	25	4	3
76	USA	35	8	1		0	1	0	0	0	0		0	1	0	500	20	3	3
77	USA	28	3.5	1		0	1	0	0	0	0					50	10	4	3
78	USA	30	3	1		0	1	0	0	0	0					60	10	3	4
79	USA	34	4	1		0	1	0	0	0	0					40	4	3	3
80	USA	27	1	1		0	1	0	0	0	0					100	25	5	4
81	USA	26	1	1		0	1	0	0	0	0					30	25	4	3
82	USA	26	2	1		0	1	0	0	0	0					50	10	4	3
83	USA	27	1	1		0	1	0	0	0	0					87.5	25	3	5
84	USA	26	3.5	1		0	0	0	0	0	1	intern				50	10	2	4
85	USA	27	0.75	1		0	1	0	0	0	0					60	5	4	2
86	USA	36	5	1		0	1	0	0	0	0					100	5	3	3
87	USA	28	1	1		0	1	0	0	0	0					60	25	4	4
88	USA	31	4	1		0	1	0	0	0	0					70	3	5	3
89	USA	33	3	1		0	1	0	0	0	0					30	10	2	3
90	USA	28	6	1		0	1	0	0	0	0					120	20	3	4
91	USA	35	5	1		0	0	0	1	0	1	pediatrics				40	10	4	3
92	USA	40	8	1		0	0	0	1	0	0					20	20	3	4
93	USA	37	8	1		0	0	0	1	0	0					30	10	4	3
94	USA	36	2	1		0	0	0	1	0	0					30	15	5	5
95	USA	36	7	1		0	1	0	0	0	0					30	5	5	5
96	USA	32	5	1		0	1	0	0	0	0					50	10	4	4
97	USA	34	5	1		0	1	0	0	0	0					50	15	3	4
98	USA	40	15	1		0	1	0	0	0	0					60	2	3	3
99	USA	40	13	1		0	1	0	0	0	0					40	10	4	4
100	USA	31	3	1		0	1	0	0	0	0					20	10	3	4
101	USA	27	2	1		0	1	0	0	0	0					60	5	4	3
102	USA	35	4	2		0	0	0	1	0	0					50	40	4	3
103	USA			1		0	0	0	1	0	0							3	4
104	USA	27	1	1		0	1	0	0	0	0					100	15	4	4
105	USA	35	5	2		0	0	0	1	0	0					75	20	3	4
106	DEU	49	19	1		1	0	1	1	1	1	Notfallmedizin	1	0	1	60	25	4	3

ID	Q1	Q2_1	Q3_1	Q4	Q4_5_othe	Q5_1	Q5_2	Q5_3	Q5_4	Q5_5	Q5_6	Q5_6_other	Q5Quota_	Q5Quota_	Q5Quota_	Q6_1	Q7_1	Q8_1	Q8_2
107	DEU	42	14	1		1	0	0	0	0	0		1	0	0	200	30	3	3
108	DEU	50	22	1		0	0	1	0	0	0		1	0	0	45	20	4	4
109	DEU	31	4	1		1	0	0	0	0	1	Innere	1	0	0	80	25	2	5
110	DEU	68	35	1		0	0	0	1	0	0		0	0	1	25	50	4	3
111	DEU	45	15	1		1	0	0	0	0	0		1	0	0	120	23	4	4
112	DEU	43	14	1		0	0	1	0	0	0		1	0	0	100	10	3	3
113	DEU	51	20	1		1	0	0	0	0	0		1	0	0	120	10	3	4
114	DEU	50	25	1		1	0	0	0	0	0		1	0	0	40	20	5	4
115	DEU	55	24	1		0	0	1	0	0	0		1	0	0	100	40	4	4
116	DEU	37	11	1		1	0	0	0	0	0		1	0	0	300	15	4	4
117	DEU	51	35	1		0	0	0	1	0	0		0	0	1	100	10	4	4
118	DEU	40	15	1		1	0	0	0	1	1	Geriatrie	1	0	0	120	15	4	4
119	DEU	53	19	1		1	0	0	0	0	0		1	0	0	80	10	4	3
120	DEU	41	11	1		1	0	0	1	0	0		1	0	1	400	10	5	4
121	DEU	45	10	1		0	0	0	1	0	0		0	0	1	150	100	5	5
122	DEU	35	8	1		0	0	1	0	0	0		1	0	0	100	40	3	5
123	DEU	37	10	1		0	0	1	0	0	1	Anästhesie / Intensivmedizin	1	0	0	200	40	3	5
124	DEU	41	14	1		0	0	0	1	0	0		0	0	1	400	10	5	3
125	DEU	42	14	1		0	0	0	1	0	0		0	0	1	80	95	4	4
126	DEU	43	14	1		1	0	0	0	0	0		1	0	0	350	35	5	4
127	DEU	49	16	1		1	1	1	1	1	0		1	1	1	500	20	4	4
128	DEU	47	14	1		1	0	0	1	1	0		1	0	1	250	30	5	3
129	DEU	40	16	1		0	0	1	0	0	0		1	0	0	200	20	5	4
130	DEU	44	17	1		1	0	0	0	0	0		1	0	0	400	20	5	4
131	DEU	48	11	1		0	1	0	0	0	0		0	1	0	60	10	5	4
132	DEU	37	8	1		0	0	0	1	0	0		0	0	1	85	30	5	3

ID	Q1	Q2_1	Q3_1	Q4	Q4_5_othe	Q5_1	Q5_2	Q5_3	Q5_4	Q5_5	Q5_6	Q5_6_other	Q5Quota_1	Q5Quota_2	Q5Quota_3	Q6_1	Q7_1	Q8_1	Q8_2
133	DEU	50	15	1		0	0	0	1	0	0		0	0	1	150	55	4	5
134	DEU	53	3	1		1	0	0	0	0	0		1	0	0	100	10	4	5
135	DEU	50	20	1		0	0	1	0	0	0		1	0	0	100	25	5	3
136	DEU	35	8	1		0	1	0	0	0	0		0	1	0	30	10	4	3
137	DEU	52	22	1		1	0	0	0	0	0		1	0	0	250	15	5	4
138	DEU	37	10	1		1	0	0	0	0	0		1	0	0	350	20	4	4
139	DEU	50	24	1		1	0	0	0	0	0		1	0	0	300	10	5	4
140	DEU	44	12	1		1	0	0	0	0	0		1	0	0	150	15	3	4
141	DEU	55	30	1		1	0	0	0	0	0		1	0	0	150	10	5	4
142	DEU	36	7	1		0	1	0	0	0	0		0	1	0	130	10	3	3
143	DEU	48	22	1		0	0	0	1	1	0		0	0	1	200	20	4	4
144	DEU	55	27	1		0	0	0	1	1	0		0	0	1	100	15	5	4
145	DEU	40	14	1		1	0	0	1	0	0		1	0	1	120	15	5	4
146	DEU	44	12	1		1	0	1	1	1	0		1	0	1	400	70	4	4
147	DEU	33	9	1		0	0	0	1	1	0		0	0	1	300	20	5	4
148	DEU	40	11	1		1	1	0	1	0	0		1	1	1	260	15	3	3
149	DEU	49	22	1		1	0	0	0	0	0		1	0	0	200	10	4	3
150	DEU	35	9	1		1	0	0	0	0	0		1	0	0	450	10	4	3
151	DEU	59	29	1		0	1	0	0	0	0		0	1	0	600	10	3	3
152	DEU	45	16	1		1	0	0	0	0	0		1	0	0	450	10	4	4
153	DEU	43	17	1		0	0	0	1	0	0		0	0	1	450	75	4	5
154	DEU	48	22	1		0	0	0	1	0	0		0	0	1	100	10	5	5
155	DEU	38	13	1		0	0	1	0	0	0		1	0	0	50	15	5	4
156	DEU	41	10	1		0	1	0	0	0	0		0	1	0	50	15	2	4
157	DEU	38	13	1		0	0	0	1	0	0		0	0	1	300	20	5	4
158	DEU	36	9	1		1	0	0	1	0	0		1	0	1	350	25	4	4
159	DEU	36	9	1		1	1	0	0	1	0		1	1	0	480	10	5	4
160	DEU	44	16	1		0	0	0	1	0	0		0	0	1	120	40	3	4
161	DEU	40	13	1		0	0	0	1	0	0		0	0	1	150	15	4	3
162	DEU	47	17	1		0	0	0	1	0	0		0	0	1	150	25	5	4
163	DEU	44	10	1		1	0	0	0	0	0		1	0	0	100	10	4	4

ID	Q1	Q2_1	Q3_1	Q4	Q4_5_othe	Q5_1	Q5_2	Q5_3	Q5_4	Q5_5	Q5_6	Q5_6_other	Q5Quota_1	Q5Quota_2	Q5Quota_3	Q6_1	Q7_1	Q8_1	Q8_2
164	DEU	49	25	1		1	0	0	0	0	0		1	0	0	350	10	5	3
165	DEU	51	23	1		1	0	0	0	0	0		1	0	0	120	15	5	4
166	DEU	51	20	1		0	0	1	0	0	0		1	0	0	50	10	4	3
167	DEU	36	8	1		1	1	0	1	1	0		1	1	1	150	20	5	3
168	DEU	40	12	1		0	0	0	1	0	0		0	0	1	350	12	5	4
169	DEU	42	13	1		0	0	0	1	1	0		0	0	1	200	15	5	4
170	DEU	54	22	1		0	1	0	1	1	0		0	1	1	35	11	4	3
171	DEU	51	19	1		0	1	0	0	0	0		0	1	0	250	15	3	3
172	DEU	41	8	1		0	1	0	0	0	0		0	1	0	30	100	4	4
173	DEU	52	20	1		0	1	0	0	0	0		0	1	0	100	10	2	3
174	DEU	35	7	1		1	1	0	0	1	0		1	1	0	80	15	5	4
175	DEU	41	9	1		0	1	0	0	0	0		0	1	0	50	10	3	4
176	DEU	48	19	1		0	1	0	0	0	0		0	1	0	400	10	2	2
177	DEU	64	35	1		0	1	0	0	0	0		0	1	0	300	10	5	5
178	DEU	47	18	1		1	1	0	1	1	0		1	1	1	200	30	3	4
179	DEU	38	9	1		0	1	0	0	0	0		0	1	0	150	40	3	4
180	DEU	37	5	1		0	1	0	0	0	0		0	1	0	200	25	5	5
181	DEU	37	9	1		0	1	0	1	1	0		0	1	1	200	10	4	3
182	USA		1	1		0	0	0	0	0	1	radiology	0	0	0	100	3	4	4
183	USA	26	1	1		0	1	0	0	0	0		0	1	0	200	5	4	4
184	USA	37	8	1		0	0	0	0	0	1	Cardiology	0	0	0	200	5	5	3
185	USA	40	12	3		0	0	1	0	0	0		0	0	1	200	10	4	3
186	USA	28	3	1		0	1	0	0	0	0		0	1	0	65	5	3	3
187	USA	44	2	1		0	1	0	0	0	0		0	1	0	70	7	3	3
188	USA	48		4		0	1	0	0	0	0		0	1	0	140	60	3	5
189	USA	37	12	1		0	0	0	0	0	0	Nephrology	0	0	0	400	20	5	5
190	USA	47	13	1	PhD	0	1	0	0	0	0		0	1	0	400	10	4	3
191	USA	32		5	MPH	0	0	0	0	0	0	Infection Prevention	0	0	0	NA	NA	NA	NA
192	USA	36	NA	5	MPH	0	0	0	0	0	0	Infection Prevention	0	0	0	NA	NA	3	3
193	USA	29	1	1		0	1	0	0	0	0		0	1	0	75	10	5	4
194	USA	32	1	2		0	1	0	0	0	0		0	1	0	65	15	3	2
195	USA	27	0.5	1		0	1	0	0	0	0		0	1	0	200	10	4	3
196	USA	30	4	1		0	0	0	0	1	0		0	0	0	10	5	4	3

ID	Q1	Q2_1	Q3_1	Q4	Q4_5_othe	Q5_1	Q5_2	Q5_3	Q5_4	Q5_5	Q5_6	Q5_6_other	Q5Quota_	Q5Quota_	Q5Quota_	Q6_1	Q7_1	Q8_1	Q8_2
197	USA	55	30	1		0	0	0	1	0	0		0	0	1	20	10	3	4
198	USA	38	8	1		0	1	0	0	0	0		0	1	0	300	7.5	5	3
199	USA	37	10	4		0	0	1	0	0	0		1	0	0	60	20	4	4
200	USA	40	3	4		0	0	1	0	0	0		1	0	0	125	50	3	5
201	USA	33	7	1		0	0	0	1	0	0		0	0	1	400	20	3	2
202	USA	39	7	1		0	1	0	0	0	0		0	1	0	100	6	3	3
203	USA	35	6	1		0	1	0	0	0	0		0	1	0	100	3	2	3
204	USA	42	18	1		0	0	0	0	0	1	Nephrology	0	0	0	400	2	5	4
205	USA	38	10	1		0	0	0	0	0	0	Nephrology	0	0	0	400	7.5	4	4
206	USA	51	20	1		0	0	1	0	0	0		1	0	0	300	15	4	3
207	USA	47	22	1		0	0	0	1	0	0		0	0	1	50	25	2	4
208	USA	34	8	2		0	0	0	1	0	0		0	0	1	30	20	3	3
209	USA	47	21	1		0	0	0	1	0	0		0	0	1	300	10	4	4
210	USA	53	27	1		0	0	0	1	0	0		0	0	1	350	5	5	2
211	USA	66	33	1		0	0	0	0	0	0	Plastics	0	0	0	50	10	5	3
212	USA	32	7	1		0	0	0	1	0	0		0	0	1	300	50	3	2
213	USA	29	4.5	1		0	1	0	0	0	0		0	1	0	275	30	4	5
214	USA	45	19	1		1	0	0	0	0	0		1	0	0	300	5	3	2
215	USA	32	5	2		0	0	0	1	0	0		0	0	1	80	33	4	4
216	USA	34	6	1		0	1	0	0	0	0		0	1	0	80	60	4	5
217	USA	31	5	2		0	0	0	1	0	0		0	0	1	300	30	3	5
218	USA	29	3	1		0	0	0	1	0	0		0	0	1	100	25	2	4
219	USA	35	4.5	1		0	0	0	1	0	1	Internal Med	0	0	1	100	20	5	4
220	USA	32	3	1		0	1	0	1	0	0		0	1	1	100	20	4	3
221	USA	34	4.5	1		0	0	0	1	0	0		0	0	1	100	45	4	3
222	USA	40	8	1		0	1	0	0	0	0		0	1	0	300	20	5	5
223	USA	34	4	1		0	0	0	1	0	0		0	0	1	100	30	4	4
224	USA	27	2	1		0	1	0	0	0	0		0	1	0	60	3	5	4
225	USA	31	3.5	1		0	0	0	1	0	0		0	0	1	100	50	4	4
226	USA	31	3.5	1		0	0	0	1	0	0		0	0	1	100	50	5	4
227	USA	52	26	1		0	0	1	0	0	0		1	0	0	200	10	3	3
228	USA	33	8	1		0	1	0	0	0	0		0	1	0	40	10	4	4
229	USA	42	8	1		0	1	0	0	0	0		0	1	0	200	20	4	4
230	USA	42	10	1		0	1	0	0	0	0		0	1	0	575	50	4	4
231	USA	71	43	1		0	0	0	0	0	0	Nephrology	0	0	0	50	10	5	5
232	USA	40	18	1		0	0	0	1	0	0		0	0	1	250	25	4	5
233	USA	31	5	1		1	0	0	0	0	0		1	0	0	250	30	4	4
234	USA	39	14	1		0	1	0	0	0	0		0	1	0	400	10	3	3
235	USA	46	20	1		0	0	0	1	0	0		0	0	1	100	35	4	3
236	USA	48	20	1		0	1	0	0	0	0		0	1	0	300	5	4	5

ID	Q1	Q2_1	Q3_1	Q4	Q4_5_othe	Q5_1	Q5_2	Q5_3	Q5_4	Q5_5	Q5_6	Q5_6_other	Q5Quota_1	Q5Quota_2	Q5Quota_3	Q6_1	Q7_1	Q8_1	Q8_2
237	USA	31	5	1		0	0	0	1	0	0		0	0	1	500	20	2	3
238	USA	62	28	2		0	0	0	1	0	0		0	0	1	200	10	3	3
239	USA	34	8	2		0	0	0	1	0	0		0	0	1	25	10	3	3
240	USA	26	2	1		0	1	0	0	0	0		0	1	0	50	10	2	4
241	USA	33	7	1		0	0	0	1	0	0		0	0	1	400	20	3	2
242	USA	33	8	1		0	0	0	1	0	0		0	0	1	800	15	5	3

ID	Q8_3	Q8_4	Q8_5	Q8_6	Q8_7	Q8_8	Q8_9	Q8_10	Q8_10_other	Q9	Q10_1	Q10_2	Q10_3	GQ11_4_o	Q11_1	Q11_2	Q11_3	Q11_4
1	2	4	3	4	4	4	3			4	100	100	50		100	0	0	
2	3	5	3	2	5	5	3			3	100	75	50		75	25	0	
3	2	5	5	3	2	3	3			4	100	75	10		100	0	0	
4	4	5	2	3	4	5	4			4	100	90	20		70	20	10	
5	3	5	3	3	3	2	3			4	100	100	90		100	0	0	
6	4	3	4	4	4	4	5			3	90	60	20		20	60	20	
7	2	4	3	3	4	3	3			4	100	95	90		100	0	0	
8	5	5	5	4	3	2	2			4	100	100	50		75	20	5	
9	4	3	3	3	2	2	3			4	100	100	100		100	0	0	
10	1	3	3	2	2	3	1			4	100	80	20		100	0	0	
11	3	3	4	2	4	4	3			4	33	33	34	Pre OP	80	5	10	5
12	2	3	3	2	3	3	3			4	100	35	10		35	35	30	
13	2	2	2	2	2	2	3			4	100	100	100		100	0	0	
14	3	4	2	2	3	1	2			4	100	80	20		80	5	15	
15	4	5	3	4	5	4	4			4	40	40	20		75	15	10	
16	2	2	3	4	4	3	3			3	80	50	50		40	50	10	
17	3	3	3	2	3	2	2			4	100	90	25		25	60	15	
18	2	3	2	2	3	2	2			4	100	100	0		80	20	0	
19	2	3	3	4	4	3	2			4	100	50	10		50	25	25	
20	3	3	3	3	3	3	3			3	100	100	50		60	20	20	
21	4	3	2	4	4	2	3			4	50	25	25		100	0	0	
22	3	5	4	4	4	3	3			4	100	100	50		100	0	0	
23	2	3	3	3	3	3	3			3	100	80	50		75	25	0	
24	3	4	2	3	3	3	2			4	100	90	90		95	5	0	

ID	Q8_3	Q8_4	Q8_5	Q8_6	Q8_7	Q8_8	Q8_9	Q8_10	Q8_10_other	Q9	Q10_1	Q10_2	Q10_3	GQ11_4_o	Q11_1	Q11_2	Q11_3	Q11_4
25	4	4	3	3	4	3	3			4	100	75	25		65	25	10	
26	4	4	2	2	3	2	2			4	100	100	75		100	0	0	
27	2	4	3	3	4	3	2			4	100	85	0		100	0	0	
28	5	2	3	1	2	3	4			4	100	100	50		100	0	0	
29	5	5	4	5	3	3	3			4	100	50	0		85	10	5	
30	5	5	4	5	5	5	4			4	100	60	20		60	30	10	
31	2	3	3	3	3	3	2			4	100	75	25		100	0	0	
32	4	5	3	3	2	2	3			4	100	100	90		100	0	0	
33	3	3	5	5	3	3	3			4	90	60	40		100	0	0	
34	5	2	3	4	2	3	4			2	30	60	10		20	60	20	
35	4	4	4	5	4	2	2			4	80	60	40		60	40	0	
36	3	3	5	3	3	3	2	2	post invasive procedure/device	4	95	50	15		75	20	5	
37	3	5	3	4	4	2	3			4	100	50	25		60	30	10	
38	2	3	2	2	4	4	3			4	100	100	80		90	0	10	
39	3	4	3	3	3	3	2			4	100	50	25		80	15	5	
40	2	4	3	3	4	3	3			4	100	70	20		80	15	5	
41	2	5	3	3	5	2	3			4	100	75	50		90	5	5	
42	3	3	3	2	3	2	2			4	100	100	60		100	0	0	
43	3	3	2	3	4	2	2			4	99	50	0		20	50	30	
44	4	4	2	3	4	3	3			4	100	50	10		95	0	5	
45	2	2	3	3	2	2	2			4	100	50	10		90	10	0	
46	2	4	3	4	4	3	3			4	100	95	40		90	10	0	
47	3	3	3	3	3	3	3			4	100	50	20		60	20	20	
48	3	5	3	3	4	4	3			3	85	65	50		100	0	0	
49	3	3	2	2	3	2	2			4	95	75	40		50	40	10	
50	4	5	4	4	2	4	5			4	70	30	0		100	0	0	

ID	Q8_3	Q8_4	Q8_5	Q8_6	Q8_7	Q8_8	Q8_9	Q8_10	Q8_10_other	Q9	Q10_1	Q10_2	Q10_3	GQ11_4_o	Q11_1	Q11_2	Q11_3	Q11_4
51	2	3	2	3	4	4	3			4	100	60	30		70	30	0	
52	1	4	2	2	5	1	2			4	80	40	0		100	0	0	
53	2	2	2	2	4	2	2	5	LVAD	4	100	60	10		60	20	20	
54	5	3	3	3	4	4	4			4	100	100	100		80	20	0	
55	2	5	3	4	5	3	2			3	100	50	0		80	0	20	
56	3	4	3	4	4	3	3			4	100	50	25		50	25	25	
57	2	4	3	4	3	3	3			4	100	90	25		95	4	1	
58	1	5	2	3	4	4	3			4	100	80	50		100	0	0	
59	3	4	5	3	3	3	4			3	50	30	20		30	30	40	
60	2	3	2	3	3	3	2			4	100	100	100		100	0	0	
61	3	4	2	3	4	3	2			4	90	10	10		100	0	0	
62	4	4	2	3	5	2	2			4	100	100	70		80	10	10	
63	3	4	3	5	4	3	2			4	100	80	30		75	15	10	
64	3	4	3	3	3	2	2			4	100	100	100		100	0	0	
65	1	2	3	2	3	3	2			4	100	90	45		100	0	0	
66	2	2	2	4	3	2	2			4	75	50	25		80	10	10	
67	2	3	3	4	2	2	2			4	100	75	10		75	15	10	
68	3	4	4	4	4	3	3			4	100	90	60		100	0	0	
69	3	2	3	3	2	2	2			3	100	80	25		100	0	0	
70	3	4	4	2	3	4	4			4	100	100	50		100	0	0	
71	2	4	3	4	4	2	3			4	100	100	0		100	0	0	
72	3	3	4	3	3	2	3			4	100	75	10		85	0	15	

ID	Q8_3	Q8_4	Q8_5	Q8_6	Q8_7	Q8_8	Q8_9	Q8_10	Q8_10_other	Q9	Q10_1	Q10_2	Q10_3	GQ11_4_o	Q11_1	Q11_2	Q11_3	Q11_4
73	3	4	3	4	5	4	4			4	100	80	30		90	10	0	
74	2	5	3	3	3	3	2			4	100	90	50		100	0	0	
75	3	4	3	5	4	4	3			4	100	98	95		95	5	0	
76	3	3	2	3	2	2	3			4	100	80	30		80	0	20	
77	2	3	2	2	3	2	2			4	100	80	60		50	30	20	
78	2	5	4	3	3	3	2			4	100	100	0		100	0	0	
79	1	2	2	3	2	3	2			4	100	100	20		80	20	0	
80	3	3	3	4	3	3	3			4	100	0	0		50	25	25	
81	3	2	2	4	3	2	1			4	100	10	0		5	15	80	
82	2	3	2	3	2	2	2			4	100	100	0		100	0	0	
83	2	3	3	3	3	3	2			4	75	50	0		50	25	25	
84	4	3	3	2	2	3	2		4 (burns pts)	4	75	50	10		50	0	50	
85	2	3	2	3	2	2	2			4	100	100	0		80	20	0	
86	3	3	2	3	2	2	2			4	100	50	10		60	40	0	
87	3	3	3	3	2	2	3			4	100	100	50		80	15	5	
88	2	3	3	4	4	2	3			4	100	50	25		80	10	10	
89	4	4	4	3	4	2	2			4	100	50	70		80	10	10	
90	2	2	3	1	1	3	3			4	80	50	10		20	80		
91	3	4	2	4	2		3			4	100	50	5		100			
92	4	4	3	3	4	4	3			4	75	50	25		50	25	25	
93	2	4	2	3	3	2	2			4	100				100			
94	2	4	5	5	5	3	5			4		100			100			
95	2	5	3	5	4	4	2			4	100	75	50		50	25	25	
96	2	3	3	3	3	4	3			4	100	75	10		20	60	20	
97	2	2	3	2	3	3	2			4	100	100	20		100			
98	2	3	2	2	3		2			4	100	80	10		30	60	10	
99	3	4	3	3	4	2	2			4	100	75	30		15	85	0	
100	4	2	2	2	4	2	5			4	100	90	10		80	20	0	0
101	4	3	2	3	4	2	5			4	100	75	20		80	10	10	0
102	2	2	2	3	2	2	2			4	95	75	50		80	0	20	0
103	3	5	3	2	2	3	2			4					100			
104	2	5	3	4	2.5		3			4	90	50	10	25	25	50	0	
105	2	4	1	2	3	2	2			4	100	100	0					
106	4	4	5	4	4	4	3			4	70	20	10		85	10	5	

ID	Q8_3	Q8_4	Q8_5	Q8_6	Q8_7	Q8_8	Q8_9	Q8_10	Q8_10_other	Q9	Q10_1	Q10_2	Q10_3	GQ11_4_o	Q11_1	Q11_2	Q11_3	Q11_4
107	3	4	3	3	3	3	3			4	100	100	10		80	0	20	
108	2	3	3	4	2	3	2			4	100	100	100		100	0	0	
109	2	3	5	1	2	5	2			4	100	100	25		60	40	0	
110	3	4	3	3	3	3	2			3	95	90	30		95	5	0	
111	4	5	4	5	5	3	3			3	100	80	60		70	25	5	
112	2	2	3	4	3	2	2			4	100	80	20		60	0	40	
113	3	3	4	3	3	3	2			3	95	80	20		75	10	15	
114	3	4	5	3	4	5	2			4	100	50	0		75	0	25	
115	4	4	4	3	3	4	3			4	100	65	0		60	10	30	
116	3	4	3	5	4	3	2			4	90	60	20		75	5	20	
117	3	3	4	3	3	3	3			3	90	70	20		80	10	10	
118	3	1	3	4	4	4	3			4	100	80	10		85	15	0	
119	3	2	3	4	3	3	3			4	100	100	0		100	0	0	
120	3	3	3	4	4	4	4			4	100	80	50		80	0	20	
121	3	4	5	5	5	5	3			4	100	40	20		90	5	5	
122	3	3	4	4	4	4	2			4	100	90	20	Empirische	50	10	10	30
123	3	5	4	5	4	2	2			4	100	100	30		80	20	0	
124	2	4	4	4	3	4	3	4	Rheumatolog. Pat. unter immunsupr. Therapie	4	100	75	60		55	35	10	
125	5	4	3	4	4	4	4			4	100	90	90		90	5	5	
126	4	4	5	3	4	4	3			4	99	50	25		50	25	25	
127	3	4	4	3	3	4	3			4	25	25	50		75	0	25	
128	3	3	4	4	4	4	4			4	100	60	20		70	20	10	
129	4	3	3	3	3	3	3			4	90	50	0		50	0	50	
130	3	3	4	4	3	4	3			4	100	100	95		100	0	0	
131	2	3	3	3	3	3	3			3	100	70	10		80	5	15	
132	3	4	3	3	2	3	2			4	99	75	50		50	30	20	

ID	Q8_3	Q8_4	Q8_5	Q8_6	Q8_7	Q8_8	Q8_9	Q8_10	Q8_10_other	Q9	Q10_1	Q10_2	Q10_3	GQ11_4_o	Q11_1	Q11_2	Q11_3	Q11_4
133	5	4	4	3	3	3	3			4	100	60	35		75	5	20	
134	3	2	5	3	3	3	2	3	Harnwegsinfekt	3	90	60	30		65	25	10	
135	3	4	4	5	3	3	2			4	100	80	30		100	0	0	
136	2	4	5	4	2	3	2			4	100	80	50		80	15	5	
137	3	4	4	4	3	4	3	5	Immunsuppression	4	100	75	20		80	15	5	
138	4	3	4	4	4	4	4			4	100	70	30		70	20	10	
139	3	4	3	4	3	3	3			4	100	90	50		100	0	0	
140	4	3	3	3	4	4	3			3	90	70	70		80	10	10	
141	3	4	3	4	4	3	4			4	100	100	100		100	0	0	
142	4	4	4	4	3	2	3			4	100	100	100		95	5	0	
143	3	4	4	3	3	4	4			3	95	80	60		70	25	5	
144	3	4	2	4	4	4	2			4	60	25	15		65	15	20	
145	3	3	4	4	2	2	3			4	100	90	70		95	0	5	
146	4	5	3	5	5	5	4			3	100	60	30		100	0	0	
147	3	3	3	4	3	3	2			3	100	80	20		25	25	50	
148	2	2	2	3	3	3	2			4	95	75	35		95	5	0	
149	3	3	3	4	3	2	3			4	100	100	50		100	0	0	
150	3	3	4	3	2	3	3			2	30	40	30		70	15	15	
151	3	3	4	4	3	4	3			2	90	70	30		90	0	10	
152	3	3	3	3	3	4	3			4	100	75	25		50	0	50	
153	4	4	4	5	4	4	4			3	85	86	85		20	30	50	
154	3	3	4	4	3	3	3			4	100	100	50		100	0	0	
155	3	4	3	4	3	2	3			4	100	80	30		75	25	0	
156	5	3	3	3	3	3	3			3	70	20	10		65	20	15	
157	2	2	2	4	3	2	2			4	100	60	10		100	0	0	
158	3	5	3	5	5	4	3			4	100	70	50		100	0	0	
159	2	3	4	3	3	3	3			4	100	99	55		95	4	1	
160	3	3	3	3	3	3	3			4	100	80	30		70	30	0	
161	3	3	4	3	4	3	2			4	100	100	30		100	0	0	
162	3	4	3	5	4	3	3			4	60	20	20		100	0	0	
163	3	2	3	4	3	2	3			3	80	10	10		100	0	0	

ID	Q8_3	Q8_4	Q8_5	Q8_6	Q8_7	Q8_8	Q8_9	Q8_10	Q8_10_other	Q9	Q10_1	Q10_2	Q10_3	GQ11_4_o	Q11_1	Q11_2	Q11_3	Q11_4
164	3	2	4	5	5	4	3			4	100	90	45		65	35	0	
165	3	4	4	4	4	3	3			4	100	75	25		100	0	0	
166	2	2	4	2	2	2	2			4	100	100	50		90	0	10	
167	2	4	3	4	3	4	3			4	100	100	90		100	0	0	
168	2	3	4	3	3	3	3			3	100	75	60		80	15	5	
169	2	3	3	4	4	2	2			4	100	80	60		100	0	0	
170	3	4	3	3	2	2	2			4	80	50	10		80	5	15	
171	3	3	3	3	3	3	3			3	90	50	20		90	0	10	
172	3	3	4	3	2	5	3			2	80	10	10		100	0	0	
173	3	2	4	3	3	3	3			3	100	80	10		90	0	10	
174	3	4	3	2	2	4	3			3	100	80	40		70	20	10	
175	3	4	4	4	3	3	2			3	100	80	50		90	0	10	
176	3	2	3	2	2	2	2			2	100	80	30		70	25	5	
177	5	5	5	5	5	4	4			4	100	80	20		100	0	0	
178	4	4	3	4	2	2	3			4	100	70	20		70	10	20	
179	4	3	5	4	2	4	4			3	100	80	20		70	20	10	
180	5	4	4	4	4	3	5			4	50	25	15		50	25	25	
181	2	4	3	3	3	4	3			4	95	75	40		40	20	40	
182	4	4	3	3	3	2	2			4	100	0	0		100	0	0	
183	4	4	2	3	4	2	4			4	100	75	10		80	10	10	
184	3	4	2	3	3	2	2			2	100	50	0		50	0	50	
185		2	2	2	3					4	50	25	5		50	0	50	
186	2	2	2	3	3	1	1	3	cellulitis	4	100	100	100		49	49	2	
187	3	3	2	3	3	4	2			4	50	15	5		100	0	0	
188	2	4	5	4	2	4	2			2	100	100	50		50	0	50	
189	2	3	3	4	4	4	2			4	100	75	25		90	0	10	
190	3	3	2	3	2	2	2			4	100	100	75		90	0	10	
191	NA	NA	NA	NA	NA	NA	NA			NA	NA	NA	NA		NA	NA	NA	
192	3	3	3	3	3	3	3			NA	NA	NA	NA		NA	NA	NA	
193	2	3	2	3	4	3	2			4	100	70	20		75	25	0	
194	1	2	3	2	3	4	2			3	90	60	20		60	30	10	
195	2	2	2	3	2	2	2			4	100				90	10	0	
196	3	2	3	3	3	2	2			4	100	100	100		100	0	0	

ID	Q8_3	Q8_4	Q8_5	Q8_6	Q8_7	Q8_8	Q8_9	Q8_10	Q8_10_other	Q9	Q10_1	Q10_2	Q10_3	GQ11_4_o	Q11_1	Q11_2	Q11_3	Q11_4
197	3	4	2	3	4	3	3			4	100	90	50		100	0	0	
198	4	4	3	4	4	2	3			4	100	100	100		100	0	0	
199	2	4	4	4	4	2	2			3	80	10	0		20	0	80	
200	2	5	2	5	5	3	3			4	100	100	50		100	0	0	
201	1	3	2	3	3	2	1			4	100	100	10		100	0	0	
202	2	4	2	3	3	2	3			4	100	100	30		100	0	0	
203	2	3	2	2	3	2	1			4	100	100	0		100	0	0	
204	3	3	3	3	3	2	2	4	IV drug users	4	100	75	25		90	5	5	
205	4	4		2	3	1	2			4	100	100	90		100	0	0	
206	3	4	2	3	4	3	2			3	100	100	100		100	0	0	
207	2	4	2	3	4	3	2			4	90	40	0		15	65	20	
208	2	2	2	2	2	2	2			4	100	75	70		95	5	0	
209	1	2	3	3	4	2	3			3	80	40	20		50	30	20	
210	2	2	2	3	3	2	2			4	100	0	0		100	0	0	
211	3	3	4	4	3	2	2			3	95	50	0		100	0	0	
212	2	4	2	3	5	2	2			4	100	100	100		90	10	0	
213	3	3	2	4	3					4	100	75	25		75	20	5	
214	2	3	2	3	3	3	2			2	100	15	0		80	15	5	
215	2	5	3	4	4	4	3			4	100	80	50		80	20	0	
216	5	5	3	4	4	3	2			4	100	80	30		90	10	0	
217	2	4	2	2	5	3	2			4	100	75	25		90	4	5	1
218	3	2	4	3	3	3	3			4	90	60	30		60	30	10	
219	2	5	3	4	4	2	2			5	100	100	80		95	5	0	
220	2	4	3	4	4	3	2			4	100	100	90		100	0	0	
221	1	4	1	2		3	2			4	100	50	10		70	30	0	
222	4	5	5	5	5	4	4			4	100	100	40		100	5	0	
223	2	4	3	3	4	2	2			4	60	40	10		90	10	0	
224	4	4	2	3	3	3	3			4	90	20	5		100	0	0	
225	3	3	3	2	4	3	2			4	100	100	50		60	20	10	10
226	3	4	3		4	3	3			4	80	10	0		100	0	0	
227	2	2	4	4	3	2	2			4	100	75	50		90	10	0	
228	3	3	2	2	3	2	2			4	100	75	10		70	20	10	
229	3	4	3	3	5	3	2			3	100	100	10		80	20	0	
230	2	4	3	4	5	3	3			4	100	80	20		80	20	0	
231	4	5	2	3	5	3	3			4	100	50	0		100	0	0	
232	4	4	3	3	4	4	3	5	tpns	4	100	80	20		90	0	10	
233	2	3	3	3	3	4	3			4	100	70	20		30	50	20	
234	4	4	4	3	4	2	3			4	100	100	0		90	10	0	
235	2	3	2	3	5	3	3			4	100	50	10		60	25	15	
236	2	3	3	4	4	3	2			4	60	30	10		100	0	0	

ID	Q8_3	Q8_4	Q8_5	Q8_6	Q8_7	Q8_8	Q8_9	Q8_10	Q8_10_other	Q9	Q10_1	Q10_2	Q10_3	GQ11_4_o	Q11_1	Q11_2	Q11_3	Q11_4
237	2	2	2	2	2	2	2			4	100	100	100		100	0	0	
238	2	2	2	3	4	2	2			4	100	100	50		50	50	0	
239	2	2	2	2	2	2	2	2		4	100	75	50		95	5	0	
240	2	4	4	2	3	3	3	4	drug users	4	50	25	25		50	35	15	
241	1	3	2	3	3	2	1			4	100	100	100		100	0	0	
242	2	5	2	2	5	2	2	2		4	100	100	0		100	0	0	

ID	Q12_1	Q12_2	Q12_3	Q12_4	Q13	Q14_1	Q14_2	Q14_3	Q14_4	Q14_4_oth	Q15_1	Q15_2	Q15_3	Q15_4	Q15_5	Q15_6	Q15_7	Q15_8	Q15_9	Q15_10
1	4	3	4	4	7	4	5	2					0	1	1	1	1	1	0	
2	3	3	2	4	7	4	4	4			1	1	1	1	1	1	1	0	1	1
3	4	4	2	3	5	4	5	3					1	1	1	1	0	1	1	0
4	5	3	4	3	7	5	5	5			1	1	0	1	1	1	1	0	1	1
5	4	2	1	5	7	5	5	5			0	0	0	1	1	1	0	1	0	0
6	3	3	3	4	1	4	4	4			1	1	1	1	1	1	0	1	1	1
7	3	1	1	3	2	5	5	4			1	1	1	1	1	1	1	1	0	0
8	3	3	2	4	2	4	3	2			0	0	1	1	0	0	1	1	1	0
9	2	3	3	3	4	3	3	3			1	1	1	0	1	1	1	1	1	1
10	4	2	3	4	4	4	4	4			1	1	1	1	1	0	0	0	1	
11	5	5	4	3	3	5	5	3			1	1	1	1	1	1	1	1	1	0
12	4	4	4	4	5	2	3	3			0	0	1	0	0	1	0	0	0	0
13	3	4	4	2	1	5	5	5			1	1	1	1	1	1	1	1	1	1
14	3	3	2	4	7	5	4	4			1	1	1	1	1	1	1	1	1	1
15	5	4	3	3	1	5	5	5			1	1	1	1	1	1	1	1	1	1
16	3	4	3	3	2	4	4	3			1	1	1	0	1	1	1	1	1	0
17	4	3	2	3	2	3	4	4			1	1	1	1	1	1	1	1	1	1
18	3	3	3	4	7	5	5	5			1	1	1	1	1	1	1	1	1	1
19	5	5	3	3	1	5	5	4			1	1	1	1	1	1	1	1	1	1
20	2	3	3	3	3	3	3	3			1	1			1	1	1	1		
21	2	4	2	4	4	4	4	4			0	0	0	1	1	1	0	0	0	0
22	3	2	2	3	1	4	5	5			1	1	1	1	1	1	1	1	1	1
23	4	2	3	3	3	4	5	4			1	1	1	0	1		1	0	1	
24	4	3	3	3	7	4	4	3			0	0	0	1	0	1	1	1	0	0

ID	Q12_1	Q12_2	Q12_3	Q12_4	Q13	Q14_1	Q14_2	Q14_3	Q14_4	Q14_4_oth	Q15_1	Q15_2	Q15_3	Q15_4	Q15_5	Q15_6	Q15_7	Q15_8	Q15_9	Q15_10
25	5	4	3	3	7	5	5	5			1	1	1	1	1	1	1	1	1	1
26	4	1	2	3	7	5	5	4			1	1	1	1	1	1	1	0	1	1
27	5	5	5	4	4	3	4	4			1	1	1	1	1	1	1	1	1	1
28	3	5	3	4	7	5	4	4			1	1	1	0	0	0	1	1	1	
29	3	4	2	5	7	3	5	5			1	1	1	1	1	1	1	1	1	1
30	4	4	5	4	7	5	5	5			1	1	1	1	1	1	1	1	1	1
31	2	2	2	5	3	5	5	5			1	1	1	0	1	1	1	1	1	1
32	3	3	2	4	3	5	5	2			0	0	0	0	0	0	0	1	0	0
33	5	5	3	4	1	5	5	2			1	1	1	0	1	1	1	1	1	1
34	3	2	4	3	3	3	2	4			1	0		1	1		1	0	0	0
35	4	4	3	3	5	4	4	3			0	0	0	1	1	1	0	1	1	
36	5	3	2	3	7	5	5	5	5	sick elderly			0	0		1		1		1
37	3	3	3	3	5	5	5	3			1	0	1	0	1	1	1	1	1	0
38	4	4	4	5	6	2	2	4			0	0		0	1	1	0	0	1	1
39	3	4	3	2	5	4	4	3			1	1	1	1	1	0	1	1	1	1
40	4	4	3	4	7	4	4	3			0	0	0	0	0		1			
41	5	3	3	5	7	4	5	4			1	1	1	1	1			1	1	1
42	4	2	1	3	7	4	5	4			1	1	1	1	1	1	1	1	1	1
43	5	4	3	4	2	5	5	5			1	1	0	0	1	1	1	1	1	1
44	3	3	2	3	7	5	5	5			1	1	1	1	1	1	1	1	1	1
45	4	3	4	5	4	3	4	5			0	0	0	1	1	1	1	1	1	0
46	5	5	4	4	2	4	4	4			1	1	1		1	1	1	1	1	1
47	3	3	3	3	7	4	4	4			1	1	1	1	1	1	1	1	1	1
48	3	3	3	3	2	5	5	5			1	1	1		1	1	1	0	1	1
49	5	3	3	3	7	3	4	4					1	1	1		0	1	1	
50	5	5	5	5	7	5	5	5			1	1	1	0	1	1	0	1	1	1

ID	Q12_1	Q12_2	Q12_3	Q12_4	Q13	Q14_1	Q14_2	Q14_3	Q14_4	Q14_4_oth	Q15_1	Q15_2	Q15_3	Q15_4	Q15_5	Q15_6	Q15_7	Q15_8	Q15_9	Q15_10
51	4	4	4	3	1	4	4	4			1	0	0	1	1	1	1	1	1	0
52	3	3	3	5	6	3	4	4			1	1	1	1	1	1		1	1	1
53	4	3	3	5	6	3	5	5			1	1	1	1	1	1	1	1	1	1
54	4	4	2	3	4	5	5	5			1	0	0	1	1	0	1	0	1	1
55	5	3	4	2	7	4	3	5			0	0	0		1			1	1	
56	3	2	3	3	5	3	4	4			1	1	1	1	1	1	1	1	1	1
57	4	1	2	2	7	5	4	4			1	1	1	1	0	0	0	0	0	0
58	5	3	3	5	7	5	5	5			1	1	1	1	1	1	1	1	1	1
59	4	4	5	4	3	4	4	5			1	0		1	1	0	0	1	0	0
60	3	3	2	3	7	4	4	4			1	1	1	1	1	1	1	1	1	1
61	4	3	3	2	2	3	4	4			1	1	1	0	1				1	1
62	3	3	3	4	6	4	5	4				1	1	0	1	1	1	1		1
63	5	2	2	4	5	4	5	4					1	0	1				1	1
64	4	2	3	5	7	5	5	5			0	0	0	0	0	0	1	1	0	0
65	4	4	3	4	7	5	5	5					0	1	1		1	1	1	
66	4	4	3	2	3	4	3	3			1	1	1	1	1	1	1	0	1	1
67	4	3	2	2	2	5	5	5					1	0	1	1	1	0	1	0
68	3	3	1	3	7	5	5	5			1	1	1		1	1	1	1	1	1
69	5	4	2	3	7	4	5	4					0	1	1	1	1	0	1	
70	4	2	2	5	3	3	3	4					1	1	1	0	0	0	1	1
71	4	4	4	4	7	4	5	3			0	0	0	0	0		0	1	0	1
72	5	5	3	3	7	4	5	5			1	1	0	0	1	1	0	0	1	1

ID	Q12_1	Q12_2	Q12_3	Q12_4	Q13	Q14_1	Q14_2	Q14_3	Q14_4	Q14_4_oth	Q15_1	Q15_2	Q15_3	Q15_4	Q15_5	Q15_6	Q15_7	Q15_8	Q15_9	Q15_10
73	5	4	5	3	5	3	5	4			1	0	1	1	1	1	1	1	1	1
74	4	3	1	2	2	5	4	4			1	1	0	1	1	1	1	1	0	1
75	4	4	3	3	1	4	5	5			1	0	0	0	1	1	1	1		0
76	4	4	4	4	1	4	4	4			1	1	1	1	1	1	1	1	1	1
77	5	3	4	4	4	5	5	5			1	1	1	1	1	1	1	1	1	1
78	5	1	2	2	7	5	5	5					1	0	1	1	1	0	1	
79	4	3	1	3	7	5	5	5			1	1	1	1	1	1	1	1	0	1
80	4	4	3	3	5	3	1	3			1		1	1	1	1	1	1	1	
81	4	1	2	2	5	4	5	1												
82	5	3	3	3	1	3	3	5			1	1	1	1	1	1	1	1	1	1
83	5	3	4	4	7	5	5	4												
84	3	2	1	3	2	4	4	2			1	1	0							
85	5	3	3	3	7	3	4	2			1	1	0	1	1	1	0	1	0	1
86	4	3	2	3	7	4	4	3			1	1	1	1	1	1	1	1	1	1
87	4	4	2	2	7	4	4	4					1		1				1	
88	4	3	3	3	6	4	4	4			1	1	0	1	1	1	1	0	0	1
89	3	3	2	4	1	5	4	4			1	1	1	1	1	1	1	1	1	1
90	3	4	3	3	7	4	2	1			1	1	1	0	1	1	1	1	1	1
91	4	2	4		5	4	4	3	2		1	1					1		1	1
92	4	1	2	4	6	3	4	4			1	1	1	1	1	1	1	1	0	0
93	5	5	5	3	7	3	4	5			1	1	1	0	1	1		1	1	1
94	5	5	5		7	5	5	5			0	0	0	1	1	1	1	1	0	0
95	5	5	5	5	7	5	5	5			1	1	1	0	0	0	0	1	0	1
96	3	4	4	3	6.5	4	4	2			1	1	1	1	1	1	1		1	1
97	3	4	3	2	7	4	5	5			0	0	0	0	0	0	0	0	0	0
98	5	3	3	3	7	5	3	4			1	1	1	1			1	1	0	0
99	4	2	2	5	7	5	4	4			0	0	1	1	1	1	1	1	0	
100	5	3	4	4	6	4	3	2	1	neutropeni	1	1	1	0	0	0	1	0	1	0
101	4	2	3	3	2	5	5	4			0	0	0	1	0	1	0	0	1	1
102	4	3	3		3	4	4	3			0	0	0	0	1	1	1	1	0	0
103	4	3			1	4	4	3		*but would still do blood cultures										
104	4	4	3	3	7	5	5	4					1	0	1	1	0	0	1	0
105	3	3	3	4	7	5	5	5			1	1	1	1	1	1	1	1	1	1
106	3	3	3	3	2	5	4	3					1		1		1		1	1

ID	Q12_1	Q12_2	Q12_3	Q12_4	Q13	Q14_1	Q14_2	Q14_3	Q14_4	Q14_4_oth	Q15_1	Q15_2	Q15_3	Q15_4	Q15_5	Q15_6	Q15_7	Q15_8	Q15_9	Q15_10
107	3	1	2	4	5	1	4	3			0	0	1	0	1	1	1	0	0	0
108	5	2	2	5	7	5	4	5			1	0	0	0	1	1		0	0	0
109	5	3	3	4	5	4	5	4			1	1	1		1			1	1	
110	4	4	3	3	5	3	4	2			1		1	0	1	1		1	1	
111	3	3	2	3	7	4	4	3			1	1	1	1	1	1	1	1	1	1
112	5	3	4	3	7	4	4	3			1	1	1	1	0	0	0	0	1	1
113	4	1	3	4	5	3	4	2			0	0	0	1	1	1	0		1	0
114	4	3	3	3	6	4	3	3			1	1	1	1	1	1	1	1	1	1
115	3	3	3	3	7	3	4	4			1	1						1	1	1
116	3	1	2	3	4	3	5	3			0	0	0	1	1	1	1	1	0	0
117	5	2	3	5	6	3	4	4			1	1	1	1	1	1		1		0
118	4	1	1	4	5	5	4	2			0	1	1	1	1	1	1	0	1	
119	3	2	2	4	6	4	4	4					1	1	1			1		
120	5	5	4	3	1	4	2	5			1	1	1	0	1	1	1	0	1	1
121	5	3	3	3	1	3	5	4			1	1	1		1	0	0	0	0	1
122	4	2	3	5	7	4	4	3												
123	4	2	3	3	5	3	4	4			1	1	1	1	1	1	1	1	1	1
124	5	4	4	4	6	4	5	4	3	Rheumatol	1	1	1		1	1	1	1	1	
125	5	5	3	2	3	4	3	5			1	1	1	0	1	1	1	0		0
126	3	3	3	3	1	5	4	4			1	1	1	0	1	0			1	1
127	4	4	4	3	2	3	4	2			1	1	1	1	1	1			1	1
128	3	3	2	4	3	5	5	5			1	1	1	1	1	0	1	1		1
129	4	2	3	3	4	2	5	4			1	1	1	1	1	1	1	0		
130	3	1	1	2	6	5	5	5			1	1	1	1	1	1	1	1		
131	4	4	3	3	7	4	4	2										1		
132	3	1	2	5	1	5	4	4			1	0	1		1	1		0	0	0

ID	Q12_1	Q12_2	Q12_3	Q12_4	Q13	Q14_1	Q14_2	Q14_3	Q14_4	Q14_4_oth	Q15_1	Q15_2	Q15_3	Q15_4	Q15_5	Q15_6	Q15_7	Q15_8	Q15_9	Q15_10
133	4	5	3	3	2	5	4	4			1	1	1	1	1	1	1	0	1	1
134	4	1	3	3	5	3	2	3			0	0	0	1	1	0	1	0	1	0
135	3	2	2	3	7	4	5	4			1	0	0	1	1	1	1	1	0	0
136	4	3	3	3	3	4	4	4					1	0	1	1	1	1	1	
137	4	5	4	3	3	4	5	3			1	1	0	0	1	0	0	1	0	0
138	3	3	3	2	6	5	5	4			0	0	0	0	1	1	1	1	0	0
139	4	3	3	4	6	3	4	4					1	1	1	1		0	1	0
140	3	4	3	3	4	3	3	4			1	0					0	0	1	1
141	3	2	3	3	5	4	5	4			0	0	1	1	0		0	1	0	0
142	4	1	3	3	1	3	4	2			0	0	0	0	0	0	0	0	0	0
143	3	4	3	4	2	4	4	2			1	1	1	0	1	1	1	1	1	1
144	3	4	3	3	5	4	2	5			0		1	1	0		0	1		0
145	2	2	1	4	7	5	5	5				0	1	1	1	1		1	1	0
146	4	2	2	3	3	5	5	5			1	1	1	1	1	1	1	1	1	1
147	3	2	4	5	2	4	5	4			1	1	1	1	1	1	1	1	1	1
148	3	3	3	3	5	3	4	4			1	1	1	1		1	1	0	1	
149	3	3	3	4	3	4	5	4			1	1	1	1	1	1	1	1	1	1
150	1	1	3	2	2	4	4	3			1	1	1	1		1	0	1	1	
151	3	3	2	3	3	4	4	4			1	1	1	1	1	1	1	1	1	1
152	4	2	3	3	3	4	4	2										1	1	0
153	4	5	5	2	3	4	5	4			1	1	1	1	1	1	1	1	1	1
154	5	4	4	3	7	3	5	4			0	1	1		1	0	0	1	1	
155	4	2	2	4	5	2	4	4			1	0	0	0	1	1	1	1	1	
156	4	3	3	2	3	4	4	3			1	0	1	1	1		1	1	0	1
157	5	5	4	5	1	4	5	3				1	1	0	1	1	1	1		1
158	4	3	5	5	3	5	5	5			1	1	1	1	1	1	1	1	1	1
159	4	3	3	3	7	4	4	4			0	0	0	0	0	0	0	1	0	0
160	4	3	3	4	5	4	4	3			1	1	1	1	1	1	1	1	1	1
161	3	2	4	4	7	4	4	3			0	0	0	1	0	0	1	1	0	1
162	3	4	2	2	1	4	5	4			1	1	0	0	0	1	1	0	0	1
163	4	3	2	3	4	3	5	2			0	0	0		0	0	0		0	1

ID	Q12_1	Q12_2	Q12_3	Q12_4	Q13	Q14_1	Q14_2	Q14_3	Q14_4	Q14_4_oth	Q15_1	Q15_2	Q15_3	Q15_4	Q15_5	Q15_6	Q15_7	Q15_8	Q15_9	Q15_10
164	4	3	2	3	7	5	5	4				0	1	1	1	1	1	1		
165	5	5	4	4	2	2	5	4			0	0	0	0	1	1	1	1		
166	4	3	3	3	5	4	5	5			1	1	1	0	1	1	1		1	0
167	3	1	2	4	5	3	4	3			1	1	1		1		1	1	1	
168	4	4	4	3	7	4	4	3			1	1	1		1	1		1	1	1
169	3	3	3	3	7	4	4	4			1	1	1	0	1	1	1	0	1	1
170	4	4	3	3	7	5	4	3					1	1	1	1				
171	5	4	3	3	3	3	3	3			1	1	1	1	1	1	1	1		1
172	5	4	5	3	3	4	3	4			1	0	1	0	0	1	0	0	0	0
173	5	5	4	3	7	5	4	4			1		1	0	1	1	1	0	1	1
174	3	4	4	4	4	3	4	5			0	0	1	0	1	0	1	0	1	0
175	4	4	3	3	3	4	4	3					1	0	1			0	0	
176	3	4	3	3	2	3	3	3				0	1	1		1	1			1
177	4	3	3	4	5	5	5	2												
178	4	2	2	3	7	4	4	4			1	1	1	1	1	1	1	1	1	1
179	3	3	4	4	2	3	4	4			1	1	1	0	1	1	1		1	1
180	5	5	3	4	2	3	4	3												
181	4	3	3	2	6	3	4	4			1	1	1	0	1	1			1	1
182	4	3	3	4	7	4	4	4			1	1	1	1	1	1	1	1	1	1
183	4	2	3	3	4	4	4	4			0	1	1	1	0	0	1	1	0	0
184	4	3	3		5	5	3	3												
185	4	3	2	?	6	4	5	3			0	1	0	1	1	1	?	1	0	1
186	3	2	2	2	6	3	4	4			0	0	0	1	1	1	1	1	1	1
187	4	4	3	3	2	4	5	4			0	0	1	1	1	1	1	1	1	0
188	2	4	3	3	4	5	4	4			1	1	0	0	0	1	1	0	1	?
189	5	5	2	4	6	5	5	3			0	0	0	0	?	1	1	1	1	0
190	4	4	3	4	2	3	4	4			1	1	1	1	1	1	1	1	1	1
191	4	3	1		4	NA	NA	NA			NA	NA	NA	NA	NA	NA	NA	NA	NA	NA
192	5	4	2	3	4	NA	NA	NA			NA	NA	NA	NA	NA	NA	NA	NA	NA	NA
193	4	5	4	4	5	4	4	3			0	0	1	1	1	?	?	1	0	0
194	5	3	4	3	4	4	4	5			1	1	1	1	1	1	1	1	1	1
195	3	3	2	4	7	4	3	3			1	1	1	?	1	1	0	?	1	?
196	4	3	2	3	7	4	4	4			1	1	1	1	1	1	1	1	1	1

ID	Q12_1	Q12_2	Q12_3	Q12_4	Q13	Q14_1	Q14_2	Q14_3	Q14_4	Q14_4_oth	Q15_1	Q15_2	Q15_3	Q15_4	Q15_5	Q15_6	Q15_7	Q15_8	Q15_9	Q15_10
197	1	1	2	3	3	3	4	3			1	1	1	0	1	0	0	1	1	1
198	5	3	1	2	2	5	5	3			1	1	1	1	1	1	1	1	1	1
199	4	4	2	4	7	5	5	5			1	1	1	1	1	1	1	1	1	1
200	5	3	3		7	5	3	4			1	1	1	?	?	?	?	1	?	
201	3	2	3	3	5	4	5	5			0	0	0	0	0	0	0	0	0	0
202	4	2	3	3	7	3	3	5		[Would nee	0	0	0	1	0	0	0	1	0	0
203	4	3	3	5	7	4	5	4			0	0	0	1	1	1	1	1	0	0
204	4	3	3	3	3	4	4	4			?	?	0	?	?	?	?	?	0	?
205	3	4	3	3	1	5	5	5			1	1	1	1	1	1	1	1	1	1
206	3	3	3	3	5	3	3	3			1	1	1	1	1	1	1	1	1	1
207	4	4	3	3	5	5	3	3	4	no explana	0	0	0	0	1	1	1	1	1	0
208	5	2	3		1	4	4	3			2	2	2	2	1	1	1	2	1	1
209	4	1	3	3	7	3	3	4			1	1	1	1	1	1	1	0	1	1
210	4	4	4	4	5	5	5	3			0	0	0	1	0	1	0	0	0	0
211	3	2	2	3	5	5	5	3			1	1	0	0	0	0	0	1	0	1
212	4	4	3	3	7	4	4	4			1	1	1	1	1	1	1	1	1	1
213	4	3	2	2	7	5	5	5			2	2	1	1	1	1	1	1	1	1
214	4	4	4	4	6	4	5	5			1	1	1	1	1	1	1	1	1	1
215	3	2	4	3	7	4	5	5			1	0	1	0	1	1	1	1	0	0
216	3	3	2		1	5	3	2			1	1	1	1	1	1	1	2	1	2
217	5	4	2	2	2	5	5	5			1	1	1	1	1	1	1	1	1	1
218	4	3	4	4	2	5	5	3			0	0	0	1	1	0	0	1	0	1
219	3	2	2	3	7	5	5	3	5	transplant	1	1	0	0	1	1	1	1	1	1
220	3	5	4	3	7	4	5	4			0	0	1	1	1	0	0	1	1	0
221	3	5	4	?	4	3	5	4			0	0	1	0	1	0	0	1	1	0
222	4	3	3	4	4	5	5	5			1	1	1	1	1	1	1	1	1	1
223	4	2	3		7	4	4	3			0	0	1	1	1	1	1	0	0	0
224	5	2	3	3	6	2	3	4			0	0	1	1	1	0	1	1	1	0
225	4	4	3	3	1	5	5	1			0	0	0	1	0	0	0	0	1	0
226	3	3	3		1	5	5	3			0	1	2	1	1	1	1	0	2	0
227	4	3	3	2	5	4	5	3			1	1	1	1	1	1	1	1	1	1
228	4	5	3	3	7	4	3	2			1	1	1	1	1	1	1	1	1	1
229	4	3	3	3	7	4	5	3			1	2	1	1	1	1	1	1	1	2
230	4	3	3	4	7	5	5	5			1	1	1	1	1	1	1	1	1	1
231	5	4	3	5	7	5	5	5			1	1	1	1	1	1	1	1	1	1
232	4	4	4	3	1	5	5	4			1	1	1	0	1	1	1	1	1	1
233	5	4	4	4	7	3	4	4			2	2	1	2	2	2	2	2	2	2
234	3	3	3	3	7	4	5	4			1	1	1	1	1	1	1	1	1	1
235	4	2	2	3	6	4	3	3			0	0	0	1	1	1	1	1	1	1
236	3	3	3	4	1	3	3	3			1	2	1	1	1	1	1	2	2	2

ID	Q12_1	Q12_2	Q12_3	Q12_4	Q13	Q14_1	Q14_2	Q14_3	Q14_4	Q14_4_oth	Q15_1	Q15_2	Q15_3	Q15_4	Q15_5	Q15_6	Q15_7	Q15_8	Q15_9	Q15_10
237	5	3	3	3	7	5	5	5			0	0	0	0	0	1	1	1	0	0
238	4	2	1	3	7	4	4	4			1	1	1	0	1	1	1	1	1	1
239	5	2	3		1	4	4	3			2	2	2	2	1	1	1	2	1	1
240	5	5	2	3	7	4	5	4			1	1	1	1	1	1	2	2	2	2
241	3	2	3	3	5	4	5	5			0	0	0	0	0	0	0	0	0	0
242	4	1	2	2	7	5	5	5			1	1	1	1	1	1	1	1	1	1

ID	Q15_11	Q15_12	Q15_13	Q15_14	Q15_15	Q15_16	Q16_1	Q16_2	Q16_3	Q16_4	Q16_5	Q16_6	Q16_7	Q16_8	Q16_9	Q17_1	Q17_2	Q17_3	Q17_4	Q17_5
1	0				1	1	1	1	1	1	1			1	1	5	5	4	5	4
2	1	1	0	0	1	1	1	0	0	1	1	0	0	0	1	3	2	3	2	3
3	1		1		1	1	1		0	1	0	0	0	0	1	4	4	4	5	5
4	1	0			1		1	1	1	1	1	1	1	1	1	5	5	5	3	5
5	0	0	0	0	1	1	1	0	0	0	1	0	0	0	1	3	3	3	2	2
6	1	1	1		1	1	1	1	1	1	1	1	1	1	1	3	4	3	4	3
7	0	0	0	0	1	1	1	0	0	0	1	0	0	0	1	3	2	2	3	2
8	0	0	0	0	1	0	0	1	1	1	0	1	1	1	1	4	5	4	2	2
9	1	1	1	1	1	1	1	1	1	1	1	1	1	1	1	3	3	4	3	3
10	1		1	1	1	0	1	1	1	1	1	1	1	1	1	4	3	3	3	3
11	1	1	1	1	1	1	1	1	1	1	1	1	1	1	1	4	4	4	3	3
12	0	0	0	0	1	1	0	1	0	1	1	1	1	1	0	3	2	3	2	3
13	1	1	1	1	1	1	1	1	0	1	1	1	1	1	1	4	4	4	4	4
14	1	1	1	1	1	1	1	1	1	1	1	1	1	1	1	5	3	3	3	2
15	1	1	1	1	1	1	1	1	1	1	1	1	1	1	1	4	4	4	4	4
16		1	1	1	1	1	1	1	1	1	0	0	1	1	0	3	4	3	2	2
17	1	1	1	1	1	1	1	1	1	1	1	1	1	1	1	2	2	2	3	2
18	1	1	1	1	1	1	1	1	1	1	1	1	1	1	1	3	3	4	3	2
19	0	0	1	0	0	1	1	1	1	1	0	0	1	1	1	5	5	5	4	4
20			1	1				1	1	1		1	1	1		2	3	3	3	2
21	1	1	1	1	0	1	1	0	1	0	0	0	0	0		4	3	3	3	2
22	1	1	1	1	1	1	1	1	1	1	1	1	1	1	1	4	4	5	4	5
23	1	1	1	1	1	1	1	1	1	1	1	1	1		1	4	4	4	4	4
24	1	0	1	0	1	1	0	1	1	1	1	1	1	1	1	2	3	1	3	4

ID	Q15_11	Q15_12	Q15_13	Q15_14	Q15_15	Q15_16	Q16_1	Q16_2	Q16_3	Q16_4	Q16_5	Q16_6	Q16_7	Q16_8	Q16_9	Q17_1	Q17_2	Q17_3	Q17_4	Q17_5
25	1	1	1	1	1	1	1	1	1	1	1	1	1	1	1	3	3	3	2	2
26	1	1	1	1	1	1	0	1	0	1	1	0	1	1	0	4	4	4	4	2
27	1	1	1	1	1	1	1	1	1	1	1	1	1	1	1	3	3	3	3	3
28	1	1	1	1		1	1	1	1	1	1	1	1	1	1	5	5	4	3	3
29	1	1	1	1	1	1	1	1	1	1	1	1	1	1	1	4	4	4	4	4
30	1	1	1	1	1	1	1	1	1	1	1	1	1	1	1	5	5	5	5	5
31	1	1	0	0	1	1	1	1	1	1	1	1	0	1	1	5	5	5	5	5
32	0	0	0	0	0	0	1	1	1	1	1	1	1	1	1	2	1	1	3	2
33	1	1	1	1	1	1	1	1	1	1	1	1	1	1	1	5	5	5	5	5
34		1	1	1	0	1	1	0	0	1	1		1	1	0	3	4	3	2	4
35	1		1	0	0		1	1	1	1		0	1		1	5	3	3	4	3
36	1	1	1	1	1		1	1	1	0	1	1	1	1	1	5	4	5	5	3
37	1	1	1	1	1	1	1	1	0	1	1	1	1	1	1	3	4	5	3	3
38	1	1	1	1	1	1	1	1	1	0	1	1	1		0	3	2	2	2	2
39	1	0	0	0	1	1	1	1	1	1	1	1	0	1	1	4	4	4	4	4
40							1	1	1	1	1	1	1	1	1	4	3	3	3	3
41	1	1	1	1			0	1	1	0	1	1	1	1	0	4	3	4	3	3
42	1	1	1	1	1	1	1	1	1	1	1	1	1	1	1	3	3	2	2	1
43	1	1	1	1	1	1	1	1	1	0	1	1	1	1	1	5	5	5	5	2
44	1	1	1	1	1	1	1	1	1	1	1	1	1	1	1	4	5	3	4	3
45	1	1	0	0	1	1	1	1	1	1	1	1	1	1	1	4	3	4	2	3
46	1	1	1	1	1	1	1	1	1	1	1	1	1	1	1	5	5	5	5	5
47	1	1	1	1	1	1	1	1	1	1	1	1	1	1	1	4	4	4	4	4
48	1	0	1	1	1	1	1	1	1	1	1	1	1	1	1	4	4	4	4	4
49	1	1	1		1	1	1	1	1				1	1	0	2	2	2	2	2
50	1	0	1	1	1	1	1	1	1	1	1	1	1	1	1	5	5	5	5	5

ID	Q15_11	Q15_12	Q15_13	Q15_14	Q15_15	Q15_16	Q16_1	Q16_2	Q16_3	Q16_4	Q16_5	Q16_6	Q16_7	Q16_8	Q16_9	Q17_1	Q17_2	Q17_3	Q17_4	Q17_5
51	1	1	1	0	1	1	1	1	1	1	1	1	1	1	1	4	3	3	3	4
52	1	1	1	1	1	1	1	1	1	1	1	1	1	1	1	3	4	3	2	3
53	1	1	1	1	1	1	1	1	1	0	1	1	1	0	1	2	2	3	3	3
54	0	0	0	0	0	0	0	0	0	0	0	0	0	0	0	2	2	2	2	2
55	0		0			0	1	1	1	0	0	1	1	1	1	4	3	3	2	2
56	1	1	1	1	1	1	1	1	1	1	1	1	1	1	1	3	4	3	2	2
57	0	0	0	0	0	0	1	1	1	1	1	1	1	1	1	3	3	3	3	3
58	1			1	1	1	1	1	1	1	1	1	1	1	1	5	5	3	3	3
59	0	0	1	0			0	0	1	1	0	0	1	1		4	4	3	3	4
60	0	0	0	1	1	1	1	1	1	1	1	1	1	1	1	3	4	4	3	3
61	1	1	1	1	1	1	1	1	1	1	1	1	1	1	1	2	2	3	3	2
62	1	1	1	1	1	1	1	1	1	0	1	1	1	1	1	4	4	2	3	2
63	1		1		1	1	1	1	1	1	0	0	1	1	1	5	4	4	4	4
64	0	0	0	0	1	0	1	1	0	1	1	0	1	1	1	2	2	3	3	3
65			0	1	1		1	1	1	1	1	0	0	1	1	4	3	4	3	2
66	1	1	0	0	0	0	1	0	0	0	1	1	1	1	0	4	4	3	5	4
67	1	1	1	0	1	1	1	1	0	1	1	1	0	0		4	5	5	5	5
68	1	1	1	1	0	1	1	1	1	0	1	1	1	1	0	4	4	5	4	4
69	0	0	0	0	1	1	1	1	0	1	1	1	1	1	1	5	3	3	4	2
70	1	1	1	0		1	0		0	0		1	1	1	1	3	3	4	3	2
71	0	0	1	1	0	1	0	1	1	0	0	1	1	1	0	4	5	3	3	4
72	1	1	0	0	1	0	1	0	1	1	1	1	0	0	1	3	3	3	3	3

ID	Q15_11	Q15_12	Q15_13	Q15_14	Q15_15	Q15_16	Q16_1	Q16_2	Q16_3	Q16_4	Q16_5	Q16_6	Q16_7	Q16_8	Q16_9	Q17_1	Q17_2	Q17_3	Q17_4	Q17_5
73	1	1	1	1	1	1	1	1	1	1	1	1	1	1	1	4	4	5	4	4
74	0	1	0	0	1	0	1	0	0	0	1	1	1	1	1	5	5	5	4	4
75	0	0	0	0	1	1	1			0	1	0	0		1	3	4	4	5	4
76	1	1	1	1	1	1	1	1	1	1	1	1	1	1	1	4	4	4	4	4
77	1	1	1	1	1	1	1	1	1	1	1	1	1	1	1	3	3	3	3	3
78	1		1		1	1	1	1	1	1	1	1				3	2	1	2	1
79	0	1	1	0	1	0	1	1	1	1	1	1	1	1	1	5	5	5	5	5
80			1		1	1	1	1	1	1	1	1	1	1	0	2	2	2	2	2
81																				
82	1	1	1	1	1	1	1	1	1	1	1	1	1	1	1	3	3	3	3	3
83																				
84						1	1	1	1	1	1	1	1	1	1	2	3	2	3	2
85	0	1	0	1	1	1	1	1	1	1	1	1	1	1	1	1	3	4	4	2
86	1	1	1	1	1	1	1	1	1	1	1	1	1	1	1	4	3	3	3	
87			1		1	1	1	1	1	1	1	1	1	1	1					
88	1	1	0	1	1	1	1	1	1	1	1	1	1	1	1	4	4	3	3	1
89	1	1	1	1	1	1	1	1	1	1	1	1	1	1	1	5	5	5	5	5
90	1	1	1	1	1	1	1	1	1	1	1	1	1	1	1	3	3	3	3	3
91	1		1				1	1	1	1		1	1	1	1	4	4	4	4	4
92	0	0	0	0	1	1	1	1	1	1	1	1	1	1	1	4	4	3	4	3
93	1	1	1	1	1	1	1	1	1	1	1	1	1	1	1	5	5	5	5	5
94	0	0	0	0	1	1	1	1	1	1	1	1	1	1	1	5	5	5	5	5
95	0	0	1	1	0	0	0	1	1	1	1	0	1	1	0	4	4	4	4	4
96	1	1		1	1	1	1	1	1	1	1	1	1	1	1	4	3	3	3	4
97	0	0	1	0	0	0	1	0	1	1	1	1	1	1	1	3	3	2	4	2
98	0	0	0	0	0	0			1	1	0	0	1	1		2	2	3	2	2
99	0	1	0	0	1	1	1	1	1	1	1	1	1	1	1	4	3	4	4	4
100	0	0	1	0	0	1	1	1	1	1	0	0	0	1	0	4	3	3	4	2
101	1	0	1	0	0	0	1	1	0	0	1	0	0	0	1	4	2	4	3	4
102	0	1	0	0	1	1	1	1	1	0	1	1	1	1	1	4	4	2	3	2
103																				
104	0		0				1	1	1	1	1			1	0	3	3	4	4	3
105	1	1	1	1	1	1	1	1	1	1	1	1	1	1	1	4	4	1	2	2
106	1	1	1	1	1	1	1	1	1	1	1	1	1	1	1	4	5	4	4	4

ID	Q15_11	Q15_12	Q15_13	Q15_14	Q15_15	Q15_16	Q16_1	Q16_2	Q16_3	Q16_4	Q16_5	Q16_6	Q16_7	Q16_8	Q16_9	Q17_1	Q17_2	Q17_3	Q17_4	Q17_5
107	1	0	1	0	0	1	0	0	0	1	1	1	0	1	1	4	4	4	3	3
108	0	0	0	0	1	1	1	1	1	1	1	1	1	1	1	2	4	2	5	3
109	1		1	0			1	0	0	0	1		0	0	1	2	4	3	4	4
110	1				1	1	1	1		1	1				0	3	2	4	3	2
111	1	1	1	1	1	1	1	1	1	1	1	1	1	1	1	4	4	3	4	4
112	1	1	1	1	0	0	1	1	1	1	1	1	1	0	1	3	4	4	4	4
113	0		0	0	1	0	0	0	0	0	1	0	0	0	1	4	5	3	4	3
114	1	1	1	1	1	0	1	0	0	0	1	1	1	0	1	4	4	4	4	4
115	1	1	1		1		1	1	1		0	1	1			3	3	4	3	2
116	0	0	1	0	1	1	0	1	1	1	0	0	0	0	1	3	4	4	4	4
117					1	1	0	1	0	1	1	0	0	1	1	2	1	2	3	1
118	1		1			1		1	1	1	1	1	0	0	0	5	4	3	5	3
119							1	1	1	1	1	1	1	1	1	3	3	3	3	3
120	1	1	1	1	0	0	1	1	1	1	1	1	1	1	0	4	5	4	4	4
121	1	0	0	0	0	1	0	1	1	1	0	1	1	1	0	3	4	4	4	4
122							1	1	1	1	1	1	1	0	0	3	3	3	4	4
123	1	1	0	0	1		1	1	1	1	1	1	1	1	1	1	2	2	3	3
124	1		1	1		1	1	1	1	1	1	1	1	1	1	4	3	3	3	3
125	1	0	0	0		0	0	1	1		0	0	0	0	1	4	3	4	4	5
126	1		1				1	1		1	1	1			1	3	3	4	4	4
127							1	1	1	1	1	1	1	1	1	4	5	4	3	4
128	1		1	1	0	0	1	1	1	1	1	1		1		4	4	4	5	4
129							1	1	1	1	1	1		1	1	4	4	4	4	4
130				0	0	1	1	1	1	1	1	1	1	1	1	5	5	4	4	3
131							1	1	1	1	1	1	1	1	1	4	4	4	4	4
132	0	0	0	0	1	1	1	0	0	0	1	0	0	0	1	4	3	2	3	2

ID	Q15_11	Q15_12	Q15_13	Q15_14	Q15_15	Q15_16	Q16_1	Q16_2	Q16_3	Q16_4	Q16_5	Q16_6	Q16_7	Q16_8	Q16_9	Q17_1	Q17_2	Q17_3	Q17_4	Q17_5
133	1		1		1	1	1	1	1	1	1	1	1	1	1	3	4	3	5	5
134	1	0	0	0	1	0	1	0	0	0	1	0	0	0	1	4	4	3	3	3
135	0	0	0	0	1		1	0	1	1	0	0	1	1	1	4	3	2	4	3
136	1	0	1				0	1	1	0	1			1	0	4	5	4	4	4
137	1	1	1	1	0	1	1	1	1	1	0	1	1	1	0	4	4	4	5	3
138	1	0	0	0	1	1	1	1	1	1	1	1	1	1	1	5	5	4	4	4
139	1	0	1			1	1		1	1	1	1		1		3	4	3	3	3
140	1	1		0			1	0	0					0	1	3	3	4	4	4
141	0	0	0	1	1	1	1	0	1	1	0	1	1	1	1	3	4	3	4	3
142	0	0	0	0	0	0	0	0	0	0	0	0	1	1	1	2	2	3	2	2
143	1	1	1	1	1	1	1	1	1	1	1	1	1	1	1	2	3	4	4	4
144	0	0	0	0		0	1	0	1	1		0	0	1	1	4	4	5	5	3
145	1	1	1	1	1	1	1	1	1	1	1	1	1	1	0	3	4	2	4	4
146	1	1	1	1	1	1	1	1	1	1	1	1	1	1	1	5	5	5	5	5
147	1	1	1	1	1	1	1	1	1	1	1	1	1	1	1	4	4	3	4	2
148	0	0	1	0	0	1	1	0	1	1	1	1	1	1	1	3	4	3	4	4
149	1	1	1	1	1	1	1	1	1	1	1	1	1	1	1	3	3	3	4	2
150	1	1	1	1	0		1	1	1	1			1	1	1	3	3	4	3	3
151	1	1	1	1	1	1	1	1	1	1	1	1	1	1	1	4	4	3	4	3
152	1		1		1	1		1	1	1	0	1	1	1	1	4	4	3	4	3
153	1	1	1	1	1	1	1	1	1	1	1	1	1	1	1	4	5	4	4	5
154	1	1	1	1	1	1	1	1	0	0	1	0	0	1	1	4	4	3	3	3
155	1	1	0	0	1		1	0	0	0	1	0	0	0	1	4	4	3	4	5
156	1	1	1	1	1	1	1	0	1	1	1	1	1	1	0	2	3	4	3	3
157	1	1	1	1		1	1	1	1	1	0	1	1	1	0	4	4	4	4	4
158	1	1	1	1	1	1	0	1	1	1	1	1	1	1	1	5	5	5	4	5
159	0	0	1	0	1	1	0	1	1	1	0		0	1	0	2	4	3	4	3
160	1	1	1	1	1	1	1	1	1	1	1	1	1	1	1	4	3	3	2	2
161	1	1	0	1	0	1	1	1	1	1	0	1	1	0	0	2	3	3	2	2
162	1	1	1	0	1	1	0	1	1	1	1	1	1			4	3	3	4	3
163	1	1	1	1	0	1	0	1	1	1	0	1	1	1	0	3	4	3	3	3

ID	Q15_11	Q15_12	Q15_13	Q15_14	Q15_15	Q15_16	Q16_1	Q16_2	Q16_3	Q16_4	Q16_5	Q16_6	Q16_7	Q16_8	Q16_9	Q17_1	Q17_2	Q17_3	Q17_4	Q17_5
164	1	1	1	1	1	1	1		1	1	1	1	1	1	1	3	4	3	5	4
165			1		1	1	1	1	0	1	1	0	0	0	1	4	4	5	4	3
166	1				1	1	1	1	1	1	1	1	1	1		3	2	4	5	2
167	1		1		1	1	1	1	1	1	1		1	1		2	2	3	5	3
168	1	1	1	1		1	1	1	1	1	1	1	1	1		4	4	3	4	3
169	1	1	1	1	1	1	0	1	1	1	0	1	1	1	0	4	4	3	4	4
170	1	1	1			1	1	1	1	1		1	1			5	4	4	3	4
171	1	1		1	1	1										3	4	4	4	4
172	0	0	1	1	1	0	0	0	0	1	1	0	0	0	0	4	3	4	5	4
173	1		1		0	1	1	1	1	1	1	1		0	0	4	4	3	4	3
174	1	1	1	1	1	1	1	1	0	0	0	1	1	0	0	3	4	3	4	2
175	1		1		1	1				1	1					2	4	3	3	3
176	1	1	1	1	1	1	1	1		1	1	1	0	0		3	3	2	3	3
177							1	1	1	1	1	1	1	1	1	5	5	4	5	5
178	1	1	1	1	1	1	1	1	1	1	1	1	1	1	1	3	4	3	4	2
179	1	1	1	1	1	1	1	1	1	1	1	1	1	1	1	3	4	4	4	3
180								1	1	1			1	1		4	4	3	4	4
181	1	1	1		1	1	1	1	1	1	1	1	1	1	1	4	4	4	5	4
182	1	1	1	1	1	1	0	1	1	1	1	1		1	1	2	2	2	2	2
183	0	1	0	0	1	0	1	1	0	1	0	1	0	0	1	3	3	4	4	3
184							1			1	1				1	4	4	2	2	3
185	1	?	0		1	1	1	1	1	1	1	1	1	1	1	4	4	4	4	3
186	0	1	0	0	1	1	1	1	1	1	1	1	1	1	1	4	4	4	4	4
187	0	0	1	0	0	1	0	1	1	1	0	0	0	1	0	3	2	4	4	2
188	1	1	1	?	1	1	1	0	1	1	0	1	?	?	?	4	3	4	4	3
189	?	?	1	0	1	1	1	0	?	0	?	?	0	0	1	2	4	3	3	3
190	1	1	1	1	1	1	1	1	1	1	1	1	1	1	1	4	4	3	3	3
191	NA	NA	NA	NA	NA	NA	NA	NA	NA	NA	NA	NA	NA	NA	NA					5
192	NA	NA	NA	NA	NA	NA	NA	NA	NA	NA	NA	NA	NA	NA	NA	5	5	5	5	5
193	0	0	0	0	0	0	1	0	0	1	1	0	0	0	1	4	4	2	2	3
194	1	1	1	1	1	1	1	1	1	1	1	1	1	1	1	3	4	5	4	3
195	1	?	1	1	?	?	1	1	1	1	1	?	?	?	?	3	2	3	2	2
196	1	1	1	1	1	1	1	1	1	1	1	1	1	1	1	4	2	3	1	1

ID	Q15_11	Q15_12	Q15_13	Q15_14	Q15_15	Q15_16	Q16_1	Q16_2	Q16_3	Q16_4	Q16_5	Q16_6	Q16_7	Q16_8	Q16_9	Q17_1	Q17_2	Q17_3	Q17_4	Q17_5
197	1	1	1	1	0	0	1	1	1	1	1	1	1	1	1	2	2	2	2	1
198	1	1	1	1	1	1	0	0	0	0	0	0	0	0	0	1	1	2	3	1
199	1	1	1	1	1	1	1	0 ?		0	1 ?		0	0	1	4	4	4	4	4
200	1 ?	?		1 ?		1 ?		1	1	1	1	1	1	1	1	4	5	3	3	4
201	0	0	0	0	0	0	1	1	1	1	1	1	1	1	1	4	3	2	3	3
202	0	0	0	0	1	1	1	1	1	0	0	1	1	1	0	3	2	2	2	1
203	0	0	0	0	1	1	1	1	1	1	1	1	1	1	1	4	3	2	2	1
204 ?	?		0 ?	?	?		0	0	0	0	0	0	0	0	0	1	2	1	1	2
205	1	1	1	1	1	1	1	1	1	1	1	1	1	1	1	3	3	3	3	3
206	1	1	1	1	1	1	1	1	1	1	1	1	1	1	1	3	3	3	3	3
207	0	2	1	0	1	1	1	1	1	0	1	1	1	1	1	5	2	3	3	3
208	1	1	2	0	1	1	1	2	1	2	1	2	0	1	1	4	4	3	4	4
209	1	1	0	0	0	1	1	1	1	0	1	0	0	0	1	4	4	3	3	3
210	0	0	0	0	0	1	1	1	0	0	1	0	0	0	1	3	2	3	3	3
211	1	1	1	1	1	1	1	1	1	1	1	1	1	1	1	4	4	4	4	4
212	1	1	1	1	1	1	1	1	1	1	1	1	1	1	1	4	4	4	4	4
213	1	2	2	2	1	1	1	1	1	1	1	1	1	1	1	5	5	3	4	4
214	1	1	1	1	1	1	1	1	1	1	1	1	1	1	1	4	4	4	5	5
215	1	1	1	1	1	1	1	1	1	1	1	1	1	1	1	5	4	4	5	3
216	1	1	1	2	2	2	1	1	1	1	1	1	2	2	2	5	5	3	4	4
217	1	1	1	1	1	1	1	1	1	1	1	1	1	1	1	2	2	4	4	4
218	0	1	1	1	1	1	1	1	1	1	1	1	0	0	1					
219	1	1	0	1	1	1	1	1	1	1	1	1	1	1	1	5	4	3	4	2
220	0	0	0	0	1	1	1	1	0	0	1	0	0	0	1	4	4	5	3	1
221	1	1	1	2	0	1	1	1	1	1	1	1	1	1	1	3	4	4	3	3
222	1	1	1	1	1	1	1	1	1	1	1	1	1	1	1	3	3	2	3	2
223	0	0	0	0	1	1	1	1	0	1	1	0	0	0	1	4	3	4	4	2
224	0	0	0	0	1	1	2	2	2	2	2	2	2	2	2	2	3	2	3	3
225	0	0	0	0	0	0	1	1	1	1	1	1	1	1	1	5	5	5	5	5
226	2	2	0	0	2	2	1	0	0	0	2	0	0	0	0	3	3	4	3	4
227	1	1	1	1	1	1	1	1	1	1	1	1	1	1	1	5	5	3	6	3
228	1	1	1	1	1	1	1	1	1	1	1	1	1	1	1	4	4	3	4	
229	1	2	1	2	1	1	1	1	1	1	1	1	1	1	1	4	4	4	4	4
230	1	1	1	1	1	1	1	1	1	1	1	1	1	1	1	5	5	5	5	5
231	1	1	1	1	1	1	1	1	1	1	1	1	1	1	1	5	5	5	5	5
232	1	1	1	1	0	1	1	1	1	1	1	1	1	1	1	5	5	5	5	5
233	1	2	1	2	1	1	1	1	1	1	1	1	2	0	0	4	4	4	3	3
234	1	1	1	1	1	1	1	1	1	1	1	1	1	1	1	4	3	3	4	4
235	1	1	0	0	1	1	1	1	1	1	1	1	1	1	1	4	3	2	3	4
236	2	2	2	2	2	2	2	2	2	1	1	2	2	2	2	4	4	4		3

ID	Q15_11	Q15_12	Q15_13	Q15_14	Q15_15	Q15_16	Q16_1	Q16_2	Q16_3	Q16_4	Q16_5	Q16_6	Q16_7	Q16_8	Q16_9	Q17_1	Q17_2	Q17_3	Q17_4	Q17_5
237	0	0	0		1	1	1	0	0	0	0	0	0	0	1	5	5	5	4	4
238	1	1	1	1	1	1	1	1	1	1	1	1	1	1	1	4	4	2	3	3
239	1	1	2	0	1	1	1	2	1	2	1	2	0	1	1	4	4	3	4	4
240	2	1	2	1	1	1	1	1	1	1	1	1	2	2	2	3	3	3	3	3
241	0	0	0	0	0	0	1	1	1	1	1	1	1	1	1	4	3	2	3	3
242	1	1	1	1	1	1	1	1	1	1	1	1	1	1	1					

ID	Q17_6	Q17_7	Q17_8	Q18_1	Q19_1	Q19_2	Q19_3	Q20	Q20v1	Q21_1	Q22_1	Q22_2	Q22_3	Q22_4
1	5	3	3	45	24	48	72	2	blood cultures always guide antibiotic choice and price is important in determined patient groups	30	1	5	4	5
2	3	3	1	50	12	24	48	1	need quicker results	25	2	2	3	2
3	4	4	4	50	3	24	72	1	Certainly choice of antibiotic changes	5	5	4	4	4
4	5	3	5	50	1	24	72	1	yes.. we tend to wait to keep broad spectrum antibiotics till culture come bACK	20	3	5	5	5
5	3	4	2	90	6	24	48	1	even current delays do prolong non-focused antibiotic therapies and discharges. but blood cultures still an essential component of care in majority of patients under scrutiny, the assay would redirect thought processes and some Rx	40	5	5	5	5
6	3	4	4	90	6	9	48	1	It does amount to additional financial burden	10	4	3	4	4
7	2	1	2	75	24	36	72	1	For certain pathogens (primarily gram positive) early results, even without susceptibility results, would allow de-escalation of antibiotic therapy	15	2	4	3	3
8	4	2	2	75	1	24	48	1	Delay in treatment while waiting for cultures can prove costly even though empiric therapy was instituted.	40	5	5	5	4
9	3	3	2	100	24	36	48	1	Susceptibility is an issue	25	2	3	3	3
10	3	2	3	90	12	24	48	2	antibiotics are started anyways and then descalated	10	3	2	2	3
11	3	2	3	90	3	24	72	2	No, I don't think so.	25	3	4	4	5
12	3	1	3	25	18	36	36	2	key is knowing the significance of an identified pathogen and where to look for source	10	2	3	3	2
13	4	4	4	70	1	24	24	2	Does Not Change My Assessment And plan	40	1	1	1	1
14	4	2	2	15	6	24	48	2	If bacteremia is suspected, the patient is placed on broad antibiotics empirically however this would help narrow the spectrum of agents quicker.	15	1	3	3	4
15	4	4	4	30	8	12	36	2	i think this is a lift threatening and benefit outweight the cost of such testing	30	4	4	4	4
16	3	3	4	60	8	20	30	2	They are often very helpful and save money allowing us to taper or narrow antibiotic spectrum	30	3	4	3	3
17	2	1	2	50	18	24	48	1	Cost related to either continuing empiric broad spectrum combination therapy pending blood culture ID and sensitivity results or to inappropriate empirical therapy with associated patient morbidity and mortality.	35	3	3	3	3
18	2	2	3	75	12	24	48	1	This is self-explanatory. It costs money for tests, meds, etc while there is a delay.	33	2	3	3	3
19	4	4	4	100	6	48	72	1	Yes, due to overuse of broad-spectrum antibiotics initially	20	3	4	4	3
20	3	3	2	80	1	2	3	1	because the patient can continue to get sicker which will cost more in the end	20	2	2	3	3
21	4	3	4	90	1	4	24	1	Antibiotic choice can make the different	50	4	4	3	3
22	4	4	4	25	12	12	24	1	Patient may have a resistant microorganism that is not being treated with the appropriate antibiotic	35	4	4	4	4
23	4	4	4	100	24	24	48	1	Relying on blood culture results definitely results in prolonged use of broad-spectrum antibiotics which are deinitely increasing the costs of these hospital stays.	75	4	4	4	4
24	3	3	3	90	6	24	48	2	Blood cultures are essential in guiding therapy	60	4	4	4	4

ID	Q17_6	Q17_7	Q17_8	Q18_1	Q19_1	Q19_2	Q19_3	Q20	Q20v1	Q21_1	Q22_1	Q22_2	Q22_3	Q22_4
25	2	1	1	50	24	24	48	1	to some extent especially when you are dealing with resistant organisms that will require a change in therapy	20	4	2	3	4
26	3	3	2	100	4	24	72	1	Too much time taken to get the results back. Broad spectrum antibiotics are expensive.	35	4	4	4	4
27	3	3	3	40	3	48	96	1	Not knowing the results of the blood culture exposes the patient to unnecessary antimicrobials.	35	4	3	3	3
28	3	3	3	25	1	1	6	1	Longer time tx with potentially inappropriate abx	25	5	4	4	4
29	4	4	4	85	12	48	72	1	You treat in the dark	20	4	4	4	4
30	5	5	5	100	24	48	72	1	Since there is a lag between specimen collection and time to positive growth, it makes a difference	25	5	5	5	5
31	5	5	5	100	12	12	24	1	Longer course emperor abx	10	4	3	3	3
32	3	1	2	20	4	12	36	1	increased resistance and medication side effects	30	3	3	3	3
33	5	5	5	70	24	48	72	2	Not at all	30	5	5	5	5
34	2	3	4	50	4	6	6	1	longer we wait longer the infection can progress	30	3	2	4	3
35	3	3	4	80	6	24	72	1	Patients may get broad-spectrum of coverage which often is not needed. These antibiotics can be more expensive. This leads to increased cost.	50	3	4	5	5
36	3	4	4	100	6	28	40	1	broader spectrum/longer duration antibiotics, potential for organ damage or adverse reactions from unnecessary atibiotics and extended stay, increase potential for resistant organisms which will require more extensive and longer duration antibiotics in future and in other patients	90	5	3	3	5
37	2	2	2	100	4	12	24	1	Yes, as the sooner you have a diagnosis, the sooner you can avoid ordering unnecessary testing, and streamline your therapy	30	4	5	4	4
38	1	2	2	10	18	36	48	1	some unnecessary antibiotic use empirically	10	2	2	3	3
39	4	3	3	50	12	24	48	1	Early goal directed therapy saves lives and money	25	4	4	4	4
40	3	3	3	20	18	18	48	1	Broader spectrum antibiotic usage	60	3	1	3	2
41	3	2	3	90	4	12	24	1	unnecessary or ineffective antibiotic therapy while awaiting culture results with direct cost and additional indirect cost related to potentially adverse patient outcomes	30	5	4	4	4
42	2	2	2	40	6	24	48	1	must use broad spectrum emperic coverage	10	3	2	2	2
43	3	2	2	80	4	24	48	1	If it is much more expensive than a blood culture the hospital may restrict its use to certain situations or not make it available at all	20	3	4	4	4
44	4	4	3	50	24	48	72	1	use of broad spectrum antibiotics rather than narrowing antibx to less costly option	25	4	3	4	4
45	4	2	1	50	4	24	48	1	Delayed de-escalation of antibiotic coverage	25	3	5	5	5
46	4	4	4	20	12	12	48	1	yes, will delay discharge patient while blood cultures are still pending	20	5	5	5	5
47	4	4	4	60	5	36	72	1	it takes up to 3 days for blood culture to help narrow the antibiotic, if that can be done sooner, patients will benefit by being treated more appropriately.	20	4	4	4	4
48	4	4	4	90	5	16	48	1	yes as we have to continue broad spectrum antibiotics atleast for 48 hours	20	3	4	4	4
49	1	1	1	20	2	27	60	1	I suppose there are both medical and finacial costs whenever there are delays for any reasons.	50	2	3	3	4
50	5	5	5	100	12	24	48	1	the faster the blood cultures are obtained and assessed, the quicker the medical decisions can be made	10	5	5	5	5

ID	Q17_6	Q17_7	Q17_8	Q18_1	Q19_1	Q19_2	Q19_3	Q20	Q20v1	Q21_1	Q22_1	Q22_2	Q22_3	Q22_4
51	4	3	3	60	10	24	48	1	may not use expensive antibiotics typically reserved for resistant org as quickly as would have been indicated.	40	2	4	4	4
52	3	3	3	60	6	24	48	1	Decisions to narrow empiric broad spectrum coverage are often delayed until final culture results are available. This is in turn leads to delays in discharges.	50	4	3	3	2
53	3	3	3	90	18	24	36	1	I don't really understand the question. There is a financial cost that stems from the increased use of broad spectrum antimicrobials. However, there is also the fact that clinical decisions about narrowing coverage cannot be made until the pathogen is known. If you are asking "Do I avoid drawing blood cultures because of their expense and do I think that negatively impact patients?" then the answer is no. Blood cultures are cheap and ordered frequently.	5	3	4	4	4
54	2	2	2	90	24	24	24	2	we are treating the patient the best way possible regardless of cost	50	4	4	4	4
55	2	3	1	40	6	24	72	1	Unnecessary use of antibiotics	50	4	4	3	3
56	2	3	3	50	6	12	24	1	Use rate of antibiotics while waiting.	30	3	3	4	3
57	4	4	3	95	2	18	48	1	Everything slower and abxs can lead to other issus	10	3	4	4	4
58	4	4	3	80	4	24	72	1	Yes, as there would be delay in narrowing the spectrum adding to the antibiotic costs, increase in resistance, and risk of Clostridium difficile infection.	40	3	5	5	5
59	4	5	4	60	6	6	8	1	Yes, the length of hospital stay is dependent on the culture results.	40	4	4	4	4
60	4	3	3	50	12	48	72	1	continue on broad spectrum antibiotics until results available	25	3	3	2	2
61	2	2	3	30	24	48	60	1	will take time to give the right antibiotic, and will make pat more complicated	20	2	3	3	3
62	3	3	2	60	18	48	72	1	Patients with suspected bloodstream infections who improve rapidly are often kept in the hospital until we get negative results after 48 or 72 hours, leading to a prolonged length of stay.	15	2	3	4	4
63	3	4	4	50	24	72	96	1	It will lead sometimes to prolongation of broad spectrum antibiotics for one, and sometimes delay appropriate Antibiotic treatment in others	45	5	5	5	5
64	2	2	2	100	24	24	48	2	I think the overall cost for treating an infection empirically, especially in cases of suspected sepsis, is the same regardless due to the overall care of the patient being the same as well as most of the advanced generation antibiotics costing relatively the same	25	2	2	3	3
65	2	2	2	100	24	24	48	1	longer time on antibiotics or higher cost broad spectrum	35	3	2	2	3
66	4	3	3	75	6	10	24	1	additional testing while you are waiting for an answer	50	4	4	4	4
67	5	5	2	100	36	36	48	1	Delay in culture results leading to delay in treatment leading to prolonged hospitalization.	50	3	4	4	5
68	4	3	4	40	24	48	56	1	expensive antibiotics, resistant organisms	40	4	4	4	4
69	2	2	4	80	4	24	48	2	Not sure if I undertanf the question	20	4	5	5	5
70	3	4	3	50	12	24	48	1	Longer stay in hospital	10	3	4	3	3
71	4	5	5	0	3	24	48	2	we overuse antibiotics currently. if a rapid system was proven to be useful less inappropriate abs would be used leading to less costly complications of ab usr ie c diff or other organ toxicity	33	1	3	4	4
72	3	3	4	75	24	24	48	1	well, we keep people on broad spectrum abx until we have a definite ID.	33	3	3	3	4

ID	Q17_6	Q17_7	Q17_8	Q18_1	Q19_1	Q19_2	Q19_3	Q20	Q20v1	Q21_1	Q22_1	Q22_2	Q22_3	Q22_4
73	4	3	3	70	8	16	30	1	Yes in some patients we might order additional diagnostic tests that are not necessarily needed if we had a rapid turnaround with the blood cx.	30	2	3	4	3
74	4	4	4	100	2	24	48	1	too long on broad spectrum abx, and even with these occasionally you may not cover the microorganism	30	5	4	4	5
75	5	5	4	50	1	24	48	1	Will prompt treaters to rely on broad spectrum empiric antibiotic regimen with potential short falls that lead to selection of more resistance, increased morbidity and mortality, as well as increased medical cost and length of stay.	5	4	5	4	4
76	4	2	3	50	6	2	10	2	Blood cultures are ordered frequently at my hospital	10	3	4	4	4
77	3	3	3	100	4	6	24	2		40	1	3	3	3
78	2	1	1	10	4	48	72	2	We over-treat already	15	5	2	2	3
79	5	5	5	80	24	24	72	1		10	5	5	5	5
80	2	2	2	10	2	24	48	2	Clinical picture/overall picture will guide ABX use, CX results only guide therapeutic choice	10	2	3	3	3
81														
82	3	3	3	10	2	12	24	1		10	3	3	3	3
83														
84	3	2	2	40	1	24	48	1	Delay in initiation of ABX for treatment proportional to time *** on the floor	75	3	4	4	4
85	3	4	4	100	24	48	72	1		30	4	3	3	3
86	3	3	3	20	24	48	72	1		40	3	3	3	2
87														
88	3	3	3	60	24	48	72	1	Delay of discharge and follow-up studies	30	3	3	3	3
89	5	5	5	75	2	24	72	1		75	5	5	5	5
90	3	3	3	80	12	36	48	2		50	3	3	3	3
91	4	4	4		9	24	108				3	4	4	4
92	3	3	3	60	12	24	72	2		25	2	4	3	3
93	5	5	5	75	24	24	60	1		50	5	5	5	5
94	3	3	3	100	4	18	72	2		30	4	3	4	3
95	4	4	4	100	24	24	120	1	Our lab turnaround time is very slow	50	4	4	4	4
96	3	4	3	50	12	12	28	1	Disposition and further treatment are delayed until final susceptibilities are received	25	4	3	3	3
97	2	3	1	100	24	36	48	1		30	1	3	3	3
98	2	1	1	30	24	48	72	1	Delay in Dx, accurate Rx, planning	20	3	4	3	4
99	4	4	4	100	24	36	60	1	Discharge hinges on that information most of the time --> increasing LOS	25	4	4	4	5
100	3	1	1	10	12	24	48	1	Prolonged hospitalization. Increased use of broad spectrum	30	3	4	3	3
101	2	3	3	50	12	36	48	2		30	3	4	4	5
102	3	4	2	50	3	12	30	1	It takes a long time to get cultures back which increases inappropriate ABX use and hospital length of stay	75	2	3	3	4
103														
104	2	3	3	90	24	48	72	1	Delay to narrow antibiotics	25	3	3	4	3
105	2	2	1	100	1	24	48	1	Empiric Abx, delay in Tx may lead to complications	20	1	4	4	4
106	4	4	4	80	2	24	36	2	Schnelle Blutkulturtests bewirkein eine Kostenreduzierung im Gesamtkontext	45	4	5	5	4

ID	Q17_6	Q17_7	Q17_8	Q18_1	Q19_1	Q19_2	Q19_3	Q20	Q20v1	Q21_1	Q22_1	Q22_2	Q22_3	Q22_4
107	2	2	2	20	24	36	48	1	Verzoegerung der Deeskalierung der ABX Therapie.	80	4	3	3	3
108	2	2	4	100	18	24	36	1	BK brauchen Zeit, bis ein Ergebnis vorliegt, deshalb muss bei V.a. Infektion immer breit antibiotisch behandelt werden. Das erhöht die Kosten.	10	4	4	4	5
109	3	3	4	20	16	36	60	2	Wir behandeln anhand der Klinik Das Ergebnis der Blutkulturen bestätigt nur das Konzept bzw macht es erforderlich es anzupassen. Aber am Anfang ist die Klinik entscheidend.	30	4	5	4	4
110	4	3	4	60	24	36	48	1	Eine deeskalation der Antibiotikatherapei wäre in vielen Fällen möglich, sobald eine genaue Identifizierung bekannt ist	20	4	4	4	3
111	4	4	3	80	12	16	30	2	Therapie wird in jedem Fall eingeleitet	70	3	3	3	4
112	4	4	3	0	24	48	72	1	unnötige breitspektrumantib	30	4	4	4	4
113	2	2	2	15	24	36	48	1	nichtindizierte Breitbandantibiotikatherapie	30	4	3	3	3
114	4	4	3	30	24	36	48	1	Nicht selten falsch negative Ergebnisse, da bereits eine antibiotische Anbehandlung stattgefunden hat.	25	4	4	4	4
115	2	2	2	10	1	24	72	2	Schnellere klinische Entscheidungen reduzieren Kosten	30	4	5	5	5
116	4	3	3	30	12	24	48	2	Bei klinischen Hinweis auf rin septisches Krankheitsbild ist eine Blutkultur obligat	50	4	4	4	5
117	2	1	2	20	18	24	36	1	Verstehe ich nicht!	30	2	3	2	2
118	4	4	5	30	8	24	36	1	je länger der test dauert desto kostenintensiver die behandlung wird (werden kann)	30	4	5	5	5
119	4	4	4	100	5	24	48	2	KOsten verzögern keine Entscheidungen - finde die Frage unverständlich	30	3	3	3	3
120	2	1	3	50	12	24	48	1	Zeitverlust bis zur Umstellung auf eine wirksame Antibiotikatherapie	40	4	4	4	4
121	4	4	4	50	24	48	72	1	ein positiver Befund muss aus forensischen Gründen eine antibiose nach sich ziehen, auch wenn es sich um eine verunreinigung handelt	40	3	4	4	4
122	4	3	3	0	2	12	24	1	Teure Antibiotikagabe	50	3	3	3	3
123	3	3	3	50	24	36	72	2	Bei Sepsisverdacht wird nach Blutkulturabnahme sofort mit der empirischen Antibiose begonnen. Daran würde auch ein Schnelltest nichts ändern.	20	4	4	4	5
124	4	2	4	50	2	6	18	1	Bis zur Identifizierung des betr. Erregers vergeht in Wirklichkeit mehr als 18 h, und das wäre noch eine optimale Zeit. Bevor das Resistogramm in verwertbarer Form vorliegt, gehen oft 24 h (++) ins Land. Da die erste Zeit "breiter" behandelt wird, kann erst nach Bekanntwerden der Ergebnisse deeskaliert werden.	45	3	4	4	4
125	3	3	2	90	1	2	3	2	Es gibt sowas bei uns nicht, natürlich es wird immer gefragt ob die Indikation stimmt	80	4	3	3	5
126	3	4	2	40	2	4	6	2	Nein, glaube ich nicht	35	3	4	4	4
127	4	4	4	100	2	4	6	2	Überall ist der Sparzwang extrem hoch.	20	5	5	5	5
128	4	5	4	70	2	18	48	2	Die Kulturtests sind preislich günstig; bei V.a. auf eine Septikämie würde definitiv ein Test laufen um Folgekosten zu senken	25	4	4	4	4
129	4	4	4	75	6	12	24	1	Zeit Verlust = Geld Verlust	50	4	4	4	4
130	3	4	5	100	24	48	72	1	Unter Umständen initial teure Breitbandantibiotika, obwohl gemäß späterem Antibiogramm Schmalspektrumantibiose möglich	30	4	4	5	4
131	4	3	4	40	8	18	96	2	da mir die Kosten des Tests nicht bekannt sind, kann ich auch keine Vermutung anstellen	50	4	4	4	4
132	3	3	3	25	17	24	48	1	Die Frage ist nicht zu verstehen	20	3	4	4	4

ID	Q17_6	Q17_7	Q17_8	Q18_1	Q19_1	Q19_2	Q19_3	Q20	Q20v1	Q21_1	Q22_1	Q22_2	Q22_3	Q22_4
133	4	4	4	50	2	8	20	2	Bluttest bewirkt eine gezielte, wirksame Therapie, die wahrscheinliche die Kosten senkt	65	3	4	4	5
134	2	2	2	30	24	36	48	1	sicherheitshalber teure Breitbandantibiotikatherapie und intensivmedizinische Behandlung	40	4	4	2	3
135	3	3	2	40	6	48	60	1	Verzögertes Erkennen resistenter Keime mit zu später effektiver Therapie. Zu späte Deeskalation bei wenig resistentem Errger, verursacht mehr Kosten und errhöht Resistenzrisiko	25	4	4	5	5
136	4	4	3	50	12	24	48	1	Fehlbehandlungen	30	4	4	4	4
137	3	3	4	60	6	12	18	1	Initiierung zu breiter Antibiosekonzepte	40	5	4	4	4
138	4	4	5	70	6	12	24	2	nein	60	4	4	4	4
139	4	3	3	35	3	24	48	1	Ergebnis und Resisitenztestung dauern zu lange, dadurch verzögerte Diagnosestellung, längere Liegezeit, höhere Kosten	30	4	4	4	5
140	3	2	3	20	12	10	6	2	keine Verzögerung	30	3	3	4	4
141	3	2	3	65	5	8	18	1	Fortführung einer unnötig breiten und relativ teuren antibiotischen Therapie	35	4	2	3	4
142	2	2	2	10	15	20	30	2	Nein, bei Verdacht auf Septikämie wird ein Breitbandantibiotikum eingesetzt, bevor das Ergebniss des BLutkulturtestes da ist. Die klinische Entscheidung kann dann im Nachhinein bei Vorliegen des Antibiogramms geändert werden.	20	3	4	4	4
143	4	3	4	60	12	12	36	1	Ja, denn während man auf das Ergebnis wartet, muss man Breit, d. h. evtl. mit überflüssigen Antibiotoka behandeln	50	3	4	4	3
144	3	3	4	20	6	24	48	1	Evtl. längerer Einsatz des nicht adaequaten Antibiotikums	25	4	4	4	4
145	5	5	5	80	3	24	72	2	Kann mir keine verzögerungen vorstellen, schnelle effiziente behandlung ist immer besser alsprimär fokus auf kostenersparnis zulegen	20	4	5	5	5
146	5	5	5	100	2	7	14	2	sehe keine Probleme	50	5	5	5	5
147	2	1	3	80	12	24	72	2	Nein	33	2	3	3	3
148	4	4	3	40	4	6	8	2	im Endeffekt kann gespart werden	50	4	4	4	4
149	2	2	2	25	4	12	24	2	Kein Kommentar	20	3	4	4	4
150	4	3	4	30	1	3	3	1	Ressourcen	15	3	2	3	4
151	3	2	3	70	4	24	36	2	Eher Kostenreduktion durch genaue Diagnostik	30	4	4	4	4
152	2	2	4	50	24	24	48	2	Mikrobiologisches Standby	25	4	4	4	3
153	2	2	5	75	1	1	1	2	da jeder Patient Anspruch auf optimale Versorgung hat	35	2	4	5	5
154	3	3	4	50	12	24	36	1	Transport und Lagerung	30	4	3	3	3
155	2	2	3	20	4	24	72	1	Bei 72 Stunden Dauer bis zur definitiven Aussage einer Blutkultur entstehen erhebliche Kosten durch die Verabreichung von Breitspektrum-ABx.Martin	25	4	4	4	5
156	4	4	3	35	2	4	2	1	Zunehmender Kostendruck im Gesundgeitswömesen	5	3	3	2	3
157	4	4	4	80	2	24	48	1	wir können ganz Einfach und schneller die Antibiotiker entscheiden	40	4	4	4	4
158	3	3	5	100	12	24	48	2	ENTSCHEDIUNGEN NICHT VON KOSTEN ABHÄNGIGI UND UMGEKEHRT	20	4	4	4	5
159	4	3	3	5	24	48	96	1	man setzt ggf. auf das falsche Antibiotikum	10	4	4	4	4
160	3	2	3	40	24	26	36	1	Deeskalation einer ggf. unnötig breiten Therapie bzw. Eskalation bei zu schmaler Therapie Insb Anpassung bei Candida	20	2	4	4	3
161	3	3	3	20	24	36	48	2	Keine spezifische Erklärung	25	3	3	3	3
162	3	3	2	75	1	12	24	1	Altes AB regime beibehalten	50	3	4	4	3
163	3	3	4	10	24	48	48	2	Untergeordnete Rolle	10	3	4	4	4

ID	Q17_6	Q17_7	Q17_8	Q18_1	Q19_1	Q19_2	Q19_3	Q20	Q20v1	Q21_1	Q22_1	Q22_2	Q22_3	Q22_4
164	5	5	5	85	12	12	48	2	Eine kalkulierte Antibiose wird immer initiiert.	35	4	4	4	3
165	3	3	3	20	8	24	28	1	Ja, da die sichere Deeskalation oder die Eskalation oft erst am 2. Tag erfolgen kann	15	3	4	4	4
166	3	3	4	70	12	24	48	1	ungenau formulierte Frage. Es entstehen Kosten -ja. Die klinische Entscheidung kann aufgrund einer Wartezeit verzögert werden - ja. Es kann sein das bei sehr teueren Entscheidungen die klinische Entscheidung hinausgezögert wird -ja.	40	4	5	4	5
167	3	3	4	75	3	8	36	2	nein	30	3	4	4	5
168	4	2	3	35	2	12	24	1	Infolge einer empirischen und oftmals mit breiterer Wirksamkeit angelegten Therapie am Anfang.	45	3	4	4	5
169	3	3	4	60	48	72	80	1	ungezielte Therapie erzeugt möglicherweise unnötige Kosten	80	4	4	3	4
170	4	3	4	60	1	12	48	2	kommt darauf an,unklare zukünftige Finanzierung	50	4	4	4	3
171	4	4	4	90	1	2	3	1	Es kostet, bestimt	30	4	4	4	4
172	3	3	4	100	1	2	3	1	Risiko minimieren	50	4	3	3	4
173	3	2	4	25	12	25	36	2	Kosten entstehen nur durch Komplikationen in der Therapie	25	2	4	4	4
174	4	4	2	20	24	72	12	1	Unerfahrenere Kollegen reagieren mit therapieumstellungen und -Eskalationen nachbresistogrammerhalt bei zb nur fakultativ Pathogenese keimen	20	3	5	5	5
175	3	3	3	20	12	24	48	1	falsche AB-Gabe	30	3	3	3	4
176	3	2	2	35	1	2	3	2	sinnvolle Kosten, denn man kommt schnell zum Ergebnis	20	3	3	2	2
177	4	4	4	100	24	24	48	1	Teure Antibiotika	70	5	5	5	5
178	3	3	3	50	18	24	36	1	erst empirische AB-Therapie bis Keim gefunden	30	3	3	2	4
179	3	2	4	70	8	24	72	1	Die Dauer der Liegezeit auf der Intensivstation kann verlaengert werden, wenn eine empirische Antibiose nicht greift, die Kulturen noch kein Ergebnis zeigen.	40	3	4	4	3
180	4	4	4	50	12	24	24	1	verlängerte diagnostik	25	3	4	4	4
181	3	2	3	30	3	8	24	1	verzögerte Behandlung, falsche Therapie, verlängerter Aufenthalt auf Intensivstation	50	3	4	4	4
182	2	2	2	50	12	24	72	1		40	3	3	3	3
183	3	4	4	100	3	24	48	1		25	1	3	4	4
184			2	5	unk	24	unk	1		?	4	2	3	3
185	4	3	4	50	4	48	48-72	1		30	3	4	4	4
186	4	4	4	5	12	18	36	1		10	4	4	3	3
187	4	3	3	30	8-Apr	12to24	24-48	1		10	3	4	4	4
188	4	4	5	50	18	24	24-48	1		75	2	4	4	4
189	4	4	4	80	18	36	72	1		25	5	4	4	4
190	3	2	2	75	6	12	48	1	Hospital days can be shortened with more rapid result times	20	4	3	4	3
191				NA	8	48	96	1		NA			3	3
192	4	3	1	NA	8	48	72	NA		NA	5	5	5	5
193	3	2	2	70	24	36	72	1		15	4	4	5	4
194	4	3	3	50	24	36	72	1		40	4	4	3	5
195	2	3	3	70	24	36	48	1		10	1	4	4	3
196	3	2	1	100	24	48	72	2		10	2	3	3	3

ID	Q17_6	Q17_7	Q17_8	Q18_1	Q19_1	Q19_2	Q19_3	Q20	Q20v1	Q21_1	Q22_1	Q22_2	Q22_3	Q22_4
197	2	2	1	10	24	48	72	2	Initiation of therapy is based mainly on clinical suspicion and risk/benefits	25	3	3	3	
198	3	3	2	100	2	24	72	1		20	3	3	3	3
199	4	4	4	30				1		10	4	4	4	4
200	4	2	2	100	24	48to72	72to120	1	Length of stay could be shortened if not having to wait for final Blood Cx results	50	4	4	5	3
201	2	1	2	50	12	24	24to48	1		25	3	5	5	5
202	2	2	2	2	12	24	48	2	I don't worry about the financial cost- just excellent patient care	5	4	2	2	2
203	3	2	1	10	0	48	72	2		5	4	2	3	3
204	2	1	2	50	8	24	72	1	Unnecessary hospital stay waiting for final results on blood culture	10	3	3	3	3
205	3	3	3	100	48	48	48to72	1		20	3	3	3	3
206	3	2	4	50	12	24	48	1		5	3	3	3	3
207	3	4	4	65	18	24	48	1		10	3	4	4	3
208	3	3	2	80	8	24	48	1	Especially for clearance of BC's. Generally do not D/C pt unless BC is negative for 24-48	70	4	3	3	3
209	2	2	2	40	12	24	48	1		50	2	3	3	3
210	3	3	3	100	0.5	24	48	1	rapid tests result in rapid responses and change of Rx	5	2	2	2	2
211	4	4	4	80	<24	>24	>24	1		33	4	4	4	4
212	4	4	4	80	12	24	48	1	reduce uncecessary tests	10	3	3	4	4
213	5	4	4	100	12-Aug	24	48	1		40	4	5	5	5
214	5	5	5	100	12	24	48	1	we may miss critical time treating wrong bact. And overuse antibiotics	10	5	5	5	5
215	3	3	3	50	14	36	72	1		25	4	4	4	4
216	3	3	4	80	24-30	>30	48	1		75	3	4	4	5
217	5	3	3	75	12	24	48	1	GPC in BC can eb a contaminant, delayed hospitalization, delayed discharge	25	2	3	4	5
218											4	4	4	4
219	5	3	2	40	24	48	72	1	Cost of unnecessary antibiiotics, increased LDS	75	4	4	5	3
220	5	4	5	30	6	36	48	1		10	5	5	5	3
221	4	3	4	50	8	24	48	1	Broad antibiotic therapy sometimes 3-4 abxs while awaiting lx results	30	2	4	4	4
222	3	3	3		48	48	48	1	Delay diagnosis	5	3	3	3	4
223	4	2	3	100	8	24	48	1			4	4	4	4
224	2	3	3	20				1		5	5	5	5	1
225	5	5	5	50	24	48	73	2		25	5	5	5	5
226	4	3	4	?	24	48	72	2		75	3	3	3	3
227	5	4	4	100	38	12	48	1		50		3	4	4
228	3	3	2	20	18	24	48	1	poor outcomes from inapproriate intial treatment	10	3	4	4	5
229	4	4	4	80	24	48	48	1	Time wasted prior to approriate treatment	20	4	3	3	3
230	5	5	5	80	24	48	72	1	Delay in treatment causes increase in morbidity and mortality as well as utilzation of resources	50	3	4	5	5
231	5	5	5	100	24	48	48	1		25	5	5	5	5
232	5	4	4	80	2	24	48	1		20	5	5	5	5
233	5	4	3	70	12	36	60	1	Delay intervention	35	3	4	4	4
234	3	3	4	75	24	48	48	1	use of antibiotics unecessarily	20	3	3	3	4
235	3	3	4	70	24	30	48	1	Gram + organisms at times cannot tell if contaminant or infection until full id known. Anerobic gram - infections can take several days to ID	15	2	3	4	4
236	4	4	4	80	12	6	24	1	Using expensive AIBs in the beginning	50	4	4	4	4

ID	Q17_6	Q17_7	Q17_8	Q18_1	Q19_1	Q19_2	Q19_3	Q20	Q20v1	Q21_1	Q22_1	Q22_2	Q22_3	Q22_4
237	4	2	5	20	24	48	72	1		50	4	4	4	4
238	3	3	3	25	12	24	48	2		10	3	3	3	3
239	3	3	2	80	10	24	48	1		70	4	3	3	3
240	3	3	3	50	12	24	48	1		25	4	4	4	4
241	2	1	2	50	12	24	48	1		25	3	5	5	5
242											3	4	4	4

ID	Q22_5	Q22_6	Q23_1	Q23_2	Q24_1	Q24_2	Q24_3	Q24_4	Q24_5	Q24_6	Q24_7	Q24_7_oth	Q25_1	Q25_2	Q25_3	Q25_4	Q25_5	Q25_5_oth	Q26
1	3	4	3	5	2	4	4	3	3	4			4	3	5	3			4
2	2	3	2	3	3	4	4	1	2	1			4	4	3	3			2
3	4	4	2	3	5	4	5	4	4	5			5	4	3	3			2
4	5	5	2	2	5	2	2	4	4	4			4	4	4	4			3
5	5	5	3	3	3	2	3	4	1	2			3	4	4	4			1
6	3	4	2	2	3	3	2	3	4	4			4	3	4	4			1
7	2	3	2	3	4	4	4	3	3	2			4	5	5	5			2
8	3	5	1	1	4	4	4	3	4	4			4	5	5	5			1
9	2	4	2	1	3	3	3	2	2	2			2	3	4	4			2
10	2	3	1	1	5	2	4	3	3	5			3	4	5	4			4
11	3	4	3	2	3	2	2	3	2	4			3	2	3	3			1
12	1	2	1	2	4	4	4	3	2	4			4	5	5	3			2
13	1	1	2	2	3	2	3	2	2	3			3	3	3	3			3
14	2	3	1	2	4	4	3	2	3	4			3	4	3	3			2
15	4	4	2	2	4	4	4	3	4	4			4	4	4	4			3
16	2	3	3	2	4	3	4	2	2	2			4	5	3	3			2
17	2	3	2	3	2	3	4	4	3	2			4	4	4	4			2
18	2	3	1	2	3	2	2	2	4	3			4	4	4	4			2
19	4	5	5	5	4	4	3	4	4	4			4	4	4	4			1
20	4	3	3	3	2	4	5	3	3	4			3	3	3	3			2
21	3	3	2	2	4	3	4	3	4	4			4	4	4	4			2
22	5	4	2	2	4	4	5	4	4	5			4	4	4	4			1
23	2	5	3	3	4	3	3	4	4	4			4	4	4	4			2
24	2	5	2	2	3	4	2	3	2	4			2	4	4	4			2

ID	Q22_5	Q22_6	Q23_1	Q23_2	Q24_1	Q24_2	Q24_3	Q24_4	Q24_5	Q24_6	Q24_7	Q24_7_oth	Q25_1	Q25_2	Q25_3	Q25_4	Q25_5	Q25_5_oth	Q26
25	2	3	1	2	3	4	4	4	4	4			4	5	4	5			2
26	3	4	1	1	5	4	4	3	2	2			4	5	5	5			3
27	3	3	1	1	3	3	3	4	4	3			4	4	4	4			4
28	4	4	1	3	2	3	4	4	4	3			3	4	4	4			4
29	4	4	2	2	4	4	4	4	4	4			4	4	4	4			2
30	5	5	3	2	4	4	4	4	4	5			5	5	4	4			2
31	1	2	2	3	3	2	3	3	3	3			3	5	5	5			4
32	3	3	1	1	3	3	3	4	3	4			4	4	5	3			2
33	5	5	1	1	5	5	5	5	5	5			1	1	5	4			4
34	2	3	3	2	3	2	3	2	3	1			3	2	3	2			2
35	3	3	3	3	4	2	3	3	2	3			5	2	4	4			3
36	4	4	4	5	3	5	5	4	5	5			5	5	5	5			2
37	2	3	2	1	4	3	4	2	1	1			4	5	5	4			1
38	2	3	1	1	2	5	5	5	4	3			5	4	4	3			4
39	4	4	2	3	3	3	3	3	3	3			4	4	4	4			3
40	2	3	1	3	1	3	5	2	2	2			5	3	5	3			4
41	4	5	5	3	4	3	3	2	2	2			4	4	4	4			2
42	1	2	2	3	4	3	3	3	1	2			4	4	4	4			2
43	4	4	2	1	4	5	2	3	3	2			4	4	4	4			2
44	3	4	2	2	3	4	4	3	4	3			4	5	5	4			1
45	2	5	1	1	4	4	5	5	3	5			4	5	5	5			1
46	4	4	2	2	5	5	5	3	3	4			4	4	4	4			3
47	4	4	2	2	4	4	4	4	4	4			4	4	4	4			3
48	4	4	1	1	3	3	3	3	3	3			4	4	4	4			3
49	2	3	3	4	3	4	4	5	5	5			4	4	5	3			3
50	5	5	3	3	5	3	4	2	3	3			5	5	2	5			1

ID	Q22_5	Q22_6	Q23_1	Q23_2	Q24_1	Q24_2	Q24_3	Q24_4	Q24_5	Q24_6	Q24_7	Q24_7_oth	Q25_1	Q25_2	Q25_3	Q25_4	Q25_5	Q25_5_oth	Q26
51	3	3	1	1	3	4	4	4	4	4			4	5	5	5			3
52	2	4	1	2	3	4	4	4	5	5			3	5	5	5			2
53	2	3	2	1	5	2	5	2	2	2			2	4	5	4			2
54	4	4	1	1	2	1	1	1	2	3			3	4	4	4			1
55	2	4	1	2	5	3	4	3	3	3			4	3	4	3			2
56	3	4	1	2	3	4	4	4	3	3			4	3	4	3			3
57	3	3	2	2	2	3	2	3	2	3			4	4	4	4			3
58	2	5	2	2	4	5	5	4	5	5			4	4	5	5			1
59	3	4	3	2	4	3	3	4	3	3			3	4	4	4			2
60	2	3	1	2	3	4	4	2	3	3			4	4	4	4			2
61	2	4	2	1	4	3	4	4	4	4			2	4	4	3			2
62	3	4	2	2	5	5	5	3	4	3			4	3	4	4			2
63	5	5	2	3	4	3	4	4	3	2			5	4	4	4			2
64	1	2	2	3	2	3	5	2	3	5			2	4	5	3			2
65	2	2	2	3	2	4	4	3	2	4			5	4	4	4			2
66	3	4	3	4	3	4	3	2	4	4			3	4	4	3			2
67	3	4	2	2	2	2	3	3	3	3			4	3	4	4			3
68	5	5	4	4	3	4	5	5	1	5			4	5	5	4			2
69	4	4	1	2	4	3	5	4	3	4			4	5	5	4			2
70	3	3	1	1	4	4	4	2	2	3			3	3	4	3			3
71	5	5	1	1	2	3	4	3	4	4			4	5	4	1			2
72	2	3	1	1	4	4	4	3	4	4			3	4	4	5			2

ID	Q22_5	Q22_6	Q23_1	Q23_2	Q24_1	Q24_2	Q24_3	Q24_4	Q24_5	Q24_6	Q24_7	Q24_7_oth	Q25_1	Q25_2	Q25_3	Q25_4	Q25_5	Q25_5_oth	Q26
73	4	3	2	3	4	3	4	3	4	5			4	4	4	4			2
74	4	3	1	2	5	4	5	5	5	4			2	5	5	5			1
75	4	5	2	3	4	4	4	5	2	4			3	3	5	4			2
76	4	4	1	2	3	4	4	4	2	3			4	3	4	4			3
77	3	3	1	2	3	4	4	4	4	4			4	4	4	3			4
78	5	5	1		1	5	5	5	5	5			1	3	4	3		Test not av	4
79	5	5	2	2	1	5	5	5	3	3			3	5	5	5			3
80	2	2	2	2	3	5	5	3	2	4			5	5	5	4			3
81																			
82	3	3	1	1	3	5	5	5	3	3			3	3	3	3			3
83																			
84	2	3	1	1	2	3	3	4	2	3			4	4	4	4			2
85	2	4	1	1	1	2	2	5	5	2			3	4	4	5			3
86	3	3	2		2	2	3	3	3	3			4	4	4	4			2
87																			
88	3	3	2	3	2	3	5	5	2	5			5	2	5	3			2
89	5	5	2	3	3	5	5	5	5	5			5	5	5	5			3
90	3	3		3	3	3	3	3	3	3			3	3	3	3			2
91	3	4			4	4	4	4	3	4			4	4	4				4
92	3	3	2	2	4	4	4	4	3	3			4	4	4				2
93	5	5	5		3	4	4	4	4	4			5	5	5	5			1
94	3	2	1	1	2	4	3	4					2	4	5	3			3
95	4	4	1	1	2	3	3	5	5	4									
96	3	4	3	2	2	4	4	4	3	5									
97	3	3	1	1	2	3	2	4	3	2									
98	3	3	1	2	3	2	3	2	3	3									
99	3	4	2	2	2	2	3	5	4	5									
100	3	4	1	1	3	4		4	3	3	4		4	4	4	3			3
101	3	3	2	3	3	4	5	5	4	3			4	3	4	2			3
102	2	3			2	3	2	5	4	2									
103													1	1	5	2			
104	4	4	2	2	2	3	4	4	4	2			4	4	5	3	3		
105	1	3	1	2	1	5	5	1	5	1			4	4	4	4	3		
106	4	4	1	1	4	3	4	3	4	4			3	4	3	3			2

ID	Q22_5	Q22_6	Q23_1	Q23_2	Q24_1	Q24_2	Q24_3	Q24_4	Q24_5	Q24_6	Q24_7	Q24_7_oth	Q25_1	Q25_2	Q25_3	Q25_4	Q25_5	Q25_5_oth	Q26
107	4	4	3	3	4	3	3	2	3	3			2	4	5	4			3
108	3	4	2	3	5	3	4	2	3	5			2	3	5	1			2
109	4	4	3	2	3	4	4	5	4	4			4	5	5	5			3
110	4	4	1	2	4	2	2	3	3	3			4	5	4	3			3
111	3	4	1	2	4	4	4	3	3	3			3	4	4	4			3
112	4	4	1	2	4	3	4	2	1	3			4	4	4	2			2
113	3	3	1	1	4	4	2	3	1	3			4	5	4	4			3
114	4	4	1	2	4	4	4	3	3	3			3	4	5	3			2
115	4	4	1	2	3	4	4	4	4	4			4	4	5	4			3
116	3	4	1	2	5	4	4	3	4	4			3	4	4	4			2
117	2	2	1	1	3	4	4	3	3	3			2	4	4	3			2
118	4	4	2	1	3	4	3	2	5	3			4	3	4	3			3
119	3	3	1	1	4	4	4	2	4	5			4	4	4	4			2
120	4	4	1	1	5	4	2	1	5	5			2	5	5	4			3
121	4	4	1	2	3	4	4	5	5	4			4	4	5	4			2
122	3	3	3	1	3	4	4	4	4	4			4	4	4	4			2
123	1	4	1	1	3	4	5	5	3	3			1	5	3	5			2
124	3	3	1	2	4	4	4	3	3	4			3	4	3	4			2
125	4	3	1	2	3	5	4	3	4	4			4	3	3	4			2
126	3	3	1	2	3	4	4	4	3	3			3	4	4	4			2
127	4	4	1	1	5	5	5	5	5	5			1	5	5	5			2
128	4	4	1	2	4	4	5	3	4	4			4	3	4	4			2
129	4	4	2	3	2	3	4	5	4	4			3	5	4	4			2
130	4	4	1	1	4	4	4	5	5	5			4	5	5	5			4
131	4	4	2	3	3	4	4	3	3	3			3	4	4	4			2
132	3	4	2	2	4	3	4	4	3	3			4	5	5	4			1

ID	Q22_5	Q22_6	Q23_1	Q23_2	Q24_1	Q24_2	Q24_3	Q24_4	Q24_5	Q24_6	Q24_7	Q24_7_oth	Q25_1	Q25_2	Q25_3	Q25_4	Q25_5	Q25_5_oth	Q26
133	4	4	3	3	3	4	4	4	5	5			3	4	4	4			2
134	2	3	1	1	4	3	2	3	2	2			4	4	4	4			3
135	4	5	2	2	5	2	4	2	3	4			2	3	5	3			2
136	4	4	1	2	1	3	3	4	4	4			4	5	4	4			2
137	3	4	1	2	5	4	4	3	4	4			2	3	5	4			2
138	4	4	1	1	4	4	4	3	4	4			3	4	4	4			2
139	3	4	1	1	4	4	3	2	3	4			3	4	4	4			2
140	3	4	1	2	3	3	4	4	3	3			3	4	2	3			1
141	3	3	1	1	4	3	5	2	5	5			4	4	5	3			2
142	4	4	2	3	4	3	4	2	4	4			2	4	4	4			2
143	3	3	1	1	4	4	4	3	2	3			3	4	4	2			2
144	4	4	1	2	4	5	4	5	5	3			4	4	4	5			3
145	5	5	1	1	3	4	4	3	4	4			2	5	3	4			1
146	5	5	1	1	5	5	5	3	4	5			4	4	3	4			1
147	2	4	1	2	3	3	4	5	4	3			4	4	2	3			2
148	4	4	1	1	4	1	1	1	1	3			3	3	3	3			2
149	4	3	1	2	3	4	4	4	4	4			3	4	4	3			3
150	4	3	2	1	3	3	3	4	3	4			3	3	2	4			1
151	3	4	3	3	4	4	4	3	3	3			2	3	2	3			2
152	2	3	1	1	4	4	4	2	3	3			3	5	4	4			3
153	5	2	5	5	4	4	4	1	4	5			3	2	4	3			1
154	3	3	1	1	4	3	3	3	3	3			3	3	3	3			3
155	4	4	1	1	3	4	3	4	4	5			2	5	2	4			2
156	3	4	2	2	4	4	3	2	2	2			3	4	4	3			2
157	4	4	1	1	4	4	4	4	4	4			4	4	4	4			1
158	3	4	1	2	3	3	4	4	3	3			3	3	3	3			1
159	4	4	1	2	4	5	3	5	3	3			2	4	4	4			2
160	4	4	1	1	4	3	4	2	4	3			2	5	5	4			2
161	3	3	1	2	4	4	5	4	5	5			3	5	4	4			2
162	3	3	2	3	4	3	3	2	3	3			4	3	4	3			1
163	3	4	2	3	4	4	3	4	3	4			3	4	5	3			1

ID	Q22_5	Q22_6	Q23_1	Q23_2	Q24_1	Q24_2	Q24_3	Q24_4	Q24_5	Q24_6	Q24_7	Q24_7_oth	Q25_1	Q25_2	Q25_3	Q25_4	Q25_5	Q25_5_oth	Q26
164	3	4	2	3	3	4	4	5	5	5			2	2	2	2			2
165	2	4	1	1	5	5	5	4	5	5			3	5	2	5			2
166	4	4	1	2	5	4	5	3	4	5			4	5	5	4			2
167	3	3	1	2	3	3	3	2	2	3			3	4	4	3			2
168	4	4	1	2	4	4	4	3	3	4			2	4	3	4			2
169	4	3	1	2	4	3	4	3	3	4			3	4	4	3			2
170	3	4	3	4	3	4	3	4	4	4			3	4	2	4			2
171	4	4	1	1	4	4	4	4	4	4			3	3	3	3			1
172	3	4	2	1	5	4	5	3	4	4			4	3	4	3			1
173	3	3	2	1	4	4	4	3	2	3			2	3	3	3			2
174	5	5	1	2	4	5	5	3	3	5			4	5	5	5			3
175	3	3	1	2	3	3	3	4	3	3			3	4	3	5			3
176	3	3	1	1	3	3	2	2	2	3			3	3	3	3			2
177	5	4	1	1	4	5	5	4	4	4			4	3	4	4			3
178	3	3	2	1	4	3	4	4	3	4			3	4	4	4			2
179	3	3	2	3	3	3	4	4	2	3			3	2	2	3			3
180	4	4	1	1	4	4	4	4	4	4			3	5	4	5			3
181	4	4	2	2	4	4	3	4	4	3			4	4	4	4			3
182	3	3	2	3	5	3	3	3	3	3			4	4	4	4			3
183	3	3	2	1	5	4	4	3	1	3			4	4	4	3			4
184	3	3	1	2	?	?	?	?	?	?			1	4	5	4			2
185	4	4	1	2	?	?	?	?	?	?			4	3	4	3			2
186	3	4	1	3	3	4	3	4	4	4			4	4	5	4			2
187	3	3		2	3	3	4	4	2	3			2	4	3	4			3
188	4	4	1	1	4	5	4	3	5	5			3	2	4	3			3
189	4	5	3	4	2	3	3	5	4	4			5	4	5	3			3
190	3	3	2	3	3	4	4	3	3	3			4	4	5	4			2
191			NA	NA	3	4	4	4		1			NA	NA	NA	NA			NA
192	5	5	NA	NA	1	4	4	5	4	3			4	4	4	4			2
193	3	5	2	3	3	4	4	3	3	4			4	4	2	4			2
194	2	3	1	2	4	3	2	2	3	3			4	5	4	5			4
195	2	3	1	1	2	4	4	3	2	4			3	4	4	3			4
196	2	2	2	1	2	4	4	4	3	3			4	4	4	2			3

ID	Q22_5	Q22_6	Q23_1	Q23_2	Q24_1	Q24_2	Q24_3	Q24_4	Q24_5	Q24_6	Q24_7	Q24_7_oth	Q25_1	Q25_2	Q25_3	Q25_4	Q25_5	Q25_5_oth	Q26
197	2	3	2	2	5	4	5	3	2	2			4	4	4	5			2
198	3	3	2	2	2	4	5	5	4	4			4	4	4	4			3
199	4	4	2	2	3	4	5	5		5			5	5	5	5			4
200	3	2	1	2	3	3	4	5		5			4	4	4	3			3
201	2	5		2	5	3	2	2	3	2			4	4	4	4			2
202	3	2	2	3	4	2	2	3	3	3			4	4	5	4			3
203	2	2	2	2	2	2	4	3	3	4			5	3	5	4			3
204	2	3	1	3	5	2	5	1	2	2			4	3	5	3			3
205	3	3	5	5	4	4	4	4	4	4			4	4	4	4			1
206	3	3	2	2	3	4	4	5	4	3			4	4	4	5			2
207	3	4	1	2	3	5	4	4	3	2									3
208	3	4	2	1	4	3	4	4	4	4			4	5	5	5			3
209	3	3	2		3	4	4	2	1	2			4	4	3	3			2
210	2	2	1	1	3	3	3	1	1	1			1	4	4	4			1
211	4	4	3	5	3	4	4	2	5	5			5	3	4	4			2
212	4	4	3	2	4	4	4	4	3	2			4	4	4				2
213	3	5	3	4	4	3	4	5	4	4			3	5	4	4			1
214	5	5	1	1	4	3	3	4	4	4			3	4	5	3			2
215	2	4	2	1	5	4	5	2	4	4			4	4	4	3			3
216	2	3	1	2	4		4						4	4	4	4			3
217	4	5	3	4	3	4	4	4	2	3			2	4	4	5			2
218	4	4	2	3	3	4	3	3	4	3			3	4	2	3			2
219	3	4	1	2	2	4	4	5	2	4			4	4	3	5			2
220	4	5	1	2	5	4	4	4	3	4			4	4	3	4			2
221	3	4	2	4	3	4	4	4	4	3			2	4	4	4			2
222	3	4	1	2	4	3	4	4	3	3			2	4	4	4			2
223	4	4	1		4	3	3	3	3	3			3	5	2	4			4
224	3	4	2		2	3	3	3	?	?			3	2	5	4			2
225	5	5	2	1	5	4	4	4	4	5			5	5	5	5			4
226	3	3	2		4	4	4	4	3	2			5	5	5	5			4
227	4	5	2	4	3	4	4	2	3	4			4	5	5	5			2
228	3	4	2	4	4	2	3	3	3	2			3	4	4	4			2
229	3	3	3	4	3	4	4	4	3	3			3	3	3	4			3
230	5	5	2	2	5	3	4	3	5	5			4	2	5	4			1
231	5	5	2	2	4	4	5	4	4	5			5	5	5	4			2
232	5	5	1	3	4	5	5	4	4	4			3	4	4	5			2
233	3	4	2	2	2	4	3	4	3	4			4	5	5	5			3
234	2	3	2	3	3	4	4	4	3	2			2	4	4	4			2
235	2	3	2	2	3	3	4	3	2	3	4		4	5	5	4			2
236	4	4	2	1	4	5	4	3	3	3			1	3	3	3			3

ID	Q22_5	Q22_6	Q23_1	Q23_2	Q24_1	Q24_2	Q24_3	Q24_4	Q24_5	Q24_6	Q24_7	Q24_7_oth	Q25_1	Q25_2	Q25_3	Q25_4	Q25_5	Q25_5_oth	Q26
237	4	4	1	1	5	5	5	5	5	5			5	4	4	4			3
238	3	4	1	2	1	5	5	4	2	3			3	4	4	4			2
239	4	2	1	4	3	4	4	4	4	4			4	5	5	5			3
240	4	4	1	1	4	3	3	3	3	5			4	2	5	2			3
241	2	5		2	5	3	2	2	3	2			4	4	4	4			2
242	3	4	3	3	4	4	4	4	3	2			4	4	4	4			2
