# Supplementary material for: Survey of Physicians’ Perspectives and Knowledge about Diagnostic Tests for Bloodstream Infections
Source: PLoS One. 2015 Mar 26;10(3):e0121493. doi: 10.1371/journal.pone.0121493 (PMC4374856; doi:10.1371/journal.pone.0121493)
Supplement: S1 Questionnaire — This is the 26 question questionnaire administered in this study. (DOCX) [file pone.0121493.s003.docx]

QUESTIONNAIRE TO UNDERSTAND MEDICAL VALUE OF TESTS TO DIAGNOSE BLOODSTREAM INFECTION

Background

1. Please select the region where you practice medicine.

USA Europe Asia/Pacific Middle East/Africa Latin America Canada

1. Please indicate your current age

_________ years.

1. Number of years you have practiced medicine since graduating medical school.

_____________years.

1. Please indicate the type of degree you hold.

MD DO PA NP Other ________.

1. Please select your medical specialty. Select all that apply.

Pulmonary/Critical Care General Medicine/Hospitalist Surgery/Surgical Critical Care

Infect. Disease/Micro Hematology/Oncology

Other (Specify)_________________________.

1. How many adult *hospital inpatients* do you see per month?

|  | Number of patients seen per month |
| --- | --- |

1. What percent of your adult *hospital inpatients* have bloodstream infections?

| % | Percent of patients with bloodstream infections |
| --- | --- |

1. In your expert opinion please rate the likelihood of each subgroup having a bloodstream infection.

| Patient subpopulation | Not at all likely | Slightly likely | Moderately likely | Very likely | Extremely likely |
| --- | --- | --- | --- | --- | --- |
| Febrile neutropenic |  |  |  |  |  |
| Critical care/Intensive care (Medical or Surgical) |  |  |  |  |  |
| Trauma |  |  |  |  |  |
| Indwelling devices (heart valves, pacemakers, tunneled catheters, grafts, etc.) |  |  |  |  |  |
| Pneumonia |  |  |  |  |  |
| Solid Organ Transplant |  |  |  |  |  |
| Hemodialysis |  |  |  |  |  |
| Older Adult |  |  |  |  |  |
| Post-Surgical |  |  |  |  |  |
| Other (specify) |  |  |  |  |  |

1. When a patient presents with a suspected bloodstream infection, how often do you order a blood culture test?

| Please Check One | Frequency of ordering blood culture |
| --- | --- |
|  | Never |
|  | Occasionally |
|  | Almost always |
|  | Always |

1. When you request a blood culture from patients *with suspec*t*ed* bloodstream infections, how often do you prescribe antibiotics empirically?

| Level of Suspicion for Bloodstream Infection | Indicate percent of patients prescribed antibiotics empirically at the same time blood culture is requested. |
| --- | --- |
| High Level of Suspicion | % |
| Medium Level of Suspicion | % |
| Low Level of Suspicion | % |

1. For patients with suspected bloodstream infections (and when the blood culture result is not yet available) indicate the treatments you generally prescribe.

| Percent of Patients Receiving the Following | Course of antibiotic treatment |
| --- | --- |
| % | Broad spectrum antibiotics |
| % | Narrow spectrum antibiotics |
| % | No antibiotics until the receipt of blood culture result |
| % | Other (specify) |
| 100% | Total |

1. Please rate certain performance characteristics of a routine blood culture.

| Performance Characteristics of a Blood Culture | Not at all acceptable | Slightly acceptable | Moderately acceptable | Very acceptable | Extremely acceptable |
| --- | --- | --- | --- | --- | --- |
| Ability to rule in an infection |  |  |  |  |  |
| Ability to rule out an infection |  |  |  |  |  |
| Turnaround time |  |  |  |  |  |
| Cost |  |  |  |  |  |

Rapid Diagnostic Test

1. Suppose a rapid diagnostic test is developed to detect bloodstream infections. This test detects the most common pathogens causing bloodstream infections, but does not provide susceptibility results.

Is there a turn-around-time at which the rapid diagnostic test for bloodstream infection would provide no additional value compared with blood culture? Select one answer.

| Please Check One | Turn Around Time of Rapid Test |
| --- | --- |
|  | Within 2 hours from clinical presentation |
|  | Within 4 hours from clinical presentation |
|  | Within 6 hours |
|  | Within 8 hours |
|  | Within 12 hours |
|  | Within 18 hours |
|  | Within 24 hours |

1. If a rapid test to detect the pathogen(s) causing bloodstream infection were available, how often would you apply its medical use for the following populations?

| Medical Indication to Apply Rapid Test | Never | Rarely | Sometimes | Often | Always |
| --- | --- | --- | --- | --- | --- |
| Any patients suspected of having bloodstream infection |  |  |  |  |  |
| High risk patients (e.g., intensive care, febrile neutropenia, etc.) |  |  |  |  |  |
| Blood culture-negative patients with moderate/high likelihood of bloodstream infection |  |  |  |  |  |
| Other Group (Explain) |  |  |  |  |  |

1. If a rapid blood test identifies one of the following organisms within hours of clinical presentation (and blood cultures are still pending), would the result lead to your narrowing the spectrum of your empiric course of antimicrobial therapy? Note: susceptibility results are unknown.

| Microorganism Identified by Rapid Test | For any of the following microorganisms, would a rapid identification result lead you to narrow the spectrum of your empiric antimicrobial coverage in the absence of susceptibility results? |
| --- | --- |
| *Enterococcus faecalis* | Yes No Uncertain |
| *Enterococcus faecium* | Yes No Uncertain |
| *Staphylococcus aureus* | Yes No Uncertain |
| *Staphylococcus epidermidis* | Yes No Uncertain |
| *Streptococcus pneumoniae* | Yes No Uncertain |
| *Streptococcus pyogenes* | Yes No Uncertain |
| *Viridans group streptococci* | Yes No Uncertain |
| *Candida albicans* | Yes No Uncertain |
| *Escherichia coli* | Yes No Uncertain |
| *Enterobacter cloacae* | Yes No Uncertain |
| *Klebsiella pneumoniae* | Yes No Uncertain |
| *Serratia marcesens* | Yes No Uncertain |
| *Pseudomonas aeruginosa* | Yes No Uncertain |
| *Acinetobacter baumanii* | Yes No Uncertain |
| *Haemophilus influenzae* | Yes No Uncertain |
| *Neisseria meningitidis* | Yes No Uncertain |

1. For each organism identified by the rapid test within hours of patient presentation, please respond if the rapid test result would change your empiric course of antimicrobial therapy.

| Microorganism Identified by Rapid Test with Susceptibility | Would a rapid result lead you to narrow or change the spectrum of antimicrobial coverage if any of the following is identified? |
| --- | --- |
| *Escherichia coli*, highly susceptible | Yes No Uncertain |
| *Enterococcus faecium*, vancomycin resistant | Yes No Uncertain |
| *Enterobacter cloacae,* ESBL producer | Yes No Uncertain |
| *Staphylococcus aureus*, methicillin-resistant | Yes No Uncertain |
| *Staphylococcus aureus,* methicillin-susceptible | Yes No Uncertain |
| *Klebsiella pneumonia*, KPC | Yes No Uncertain |
| *Acinetobacter baumanii* (highly resistant) | Yes No Uncertain |
| *Candida glabrata* (fluconazole resistant) | Yes No Uncertain |
| *Candida albicans* (fluconazole susceptible) | Yes No Uncertain |

1. Please rate the level of impact of a rapid diagnostic test (result within hours) compared with routine blood culture for bloodstream infection on clinical practices.

| Influence of a Rapid Test Result on Clinical Practices | Not at all impact | Slightly impact | Moderately impact | Very impactful | Extremely impactful |
| --- | --- | --- | --- | --- | --- |
| Reduce overall antibiotic consumption |  |  |  |  |  |
| Decrease emergence of antimicrobial resistance |  |  |  |  |  |
| Reduce intensity of healthcare utilization (i.e. imaging studies, other blood tests, specialty consults, etc.) |  |  |  |  |  |
| Decrease patient morbidity and mortality |  |  |  |  |  |
| Reduce infection transmission to other susceptible patients |  |  |  |  |  |
| Reduce overall costs of hospitalization |  |  |  |  |  |
| Payor satisfaction |  |  |  |  |  |
| Patient satisfaction |  |  |  |  |  |

1. If a rapid test for bloodstream infection were available without susceptibility testing, what percent of your patients would you test?

| % | What percent of your patients would you test with a rapid test? |
| --- | --- |

1. What is the typical turnaround time in your institution between collecting a culture and the following (in hours)?

| Hrs | Gram stain result reported to you/unit from instrument flagged blood culture bottle |
| --- | --- |
| Hrs | Preliminary identification of pathogen from blood culture |
| Hrs | Final identification of pathogen with susceptibility results from blood culture |

1. In your expert medical opinion, do you think there is a financial cost from relying on blood culture results that can potentially lead to a delay in clinical decisions?

| Please Check One |  |
| --- | --- |
|  | Yes |
|  | No |

Please explain:

|  |
| --- |

1. In your expert medical opinion, what percent of patients are treated with an incorrect or suboptimal antibiotic/antifungal as the physician awaits blood culture results?

| % | Percent of patients treated with incorrect or suboptimal antibiotic/antifungal regimen |
| --- | --- |

1. We are interested in understanding your perspective on how incorrect or suboptimal antimicrobial regimens may affect the course of a patient’s hospitalization in terms of outcomes and cost. Please rate how you think a delay in initiating optimal antimicrobial therapy would impact the following variables.

|  | No impact | Slightly impact | Moderately impact | Highly impact | Extremely High  impact |
| --- | --- | --- | --- | --- | --- |
| Cost of antibiotic regimen |  |  |  |  |  |
| Increase days in ICU |  |  |  |  |  |
| Increase length of stay of total hospitalization |  |  |  |  |  |
| Increase patient morbidity or mortality |  |  |  |  |  |
| Increase staff time (i.e. increase work load for nurse, tech or physician) |  |  |  |  |  |
| Total cost of hospitalization |  |  |  |  |  |

1. At what maximum price point would you be willing to order a rapid test that could provide results within hours –and as an adjunct to standard of care blood culture?

| Maximum Price Point for Rapid Test | $100 | $200 | $400 | $600 | $800 |
| --- | --- | --- | --- | --- | --- |
| Test used as adjunct to standard of care blood culture |  |  |  |  |  |
| Rapid test used as stand alone |  |  |  |  |  |

1. Please rate the level of influence for each decision maker when your institution decides to adopt a new rapid test to detect bloodstream infections.

| Decision-Maker | Not at all influential | Slightly influential | Moderately influential | Very influential | Extremely influential |
| --- | --- | --- | --- | --- | --- |
| Clinicians |  |  |  |  |  |
| Laboratory Administrator (of all labs) |  |  |  |  |  |
| Physician or PhD Microbiology Medical Director |  |  |  |  |  |
| Hospital Administrators |  |  |  |  |  |
| P&T committee or other similar committee |  |  |  |  |  |
| Infection control/hospital epidemiology committee |  |  |  |  |  |
| Other (specify) |  |  |  |  |  |

1. Given certain level of test performance, please rate the obstacles to adoption of a rapid test.

| Obstacles to Adoption of Rapid Test | Strongly Disagree | | Disagree | Neutral | Agree | Strongly Agree |
| --- | --- | --- | --- | --- | --- | --- |
| Physician uncertainty of using new technology in clinical practice |  | |  |  |  |  |
| Cost of Test |  | |  |  |  |  |
| Lack of evidence for clinical utility |  | |  |  |  |  |
| No reimbursement code or payor concerns |  | |  |  |  |  |
| Other Obstacles (Specify): | |  | | | | |

1. When a new test, technology, or procedure becomes available for use on your patients, are you more likely to implement early on, or wait until it has become more established?

| Please Check One |  |
| --- | --- |
|  | Earliest adopters/risk taker |
|  | Early adopter |
|  | Adopt after established with majority of peers |
|  | Wait until well-tested in field and many published studies available |
